# Irradiated Tumor Cell‐Derived Microparticles Activate Systemic Anti‐Tumor Immunity via the STING/NLRP3/GSDMD Axis in Neutrophils

**DOI:** 10.1002/advs.202514390

**Published:** 2026-01-04

**Authors:** Yan Hu, Jiacheng Wang, Mengjie Che, Zheng Yang, Jingshu Meng, Xiao Yang, Yue Deng, Zhiyuan Zhou, Yijun Wang, Wenwen Wei, Zhanjie Zhang, Bian Wu, You Qin, Kunyu Yang, Honglin Jin, Fang Huang, Yajie Sun, Lu Wen, Chao Wan

**Affiliations:** ^1^ Cancer Center Union Hospital Tongji Medical College Huazhong University of Science and Technology Wuhan China; ^2^ Institute of Radiation Oncology Union Hospital Tongji Medical College Huazhong University of Science and Technology Wuhan China; ^3^ Hubei Key Laboratory of Precision Radiation Oncology Wuhan China; ^4^ Ministry of Education Key Laboratory of Biological Targeted Therapy (Huazhong University of Science and Technology Wuhan Hubei China; ^5^ College of Biomedicine and Health and College of Life Science and Technology Huazhong Agricultural University Wuhan China

**Keywords:** extracellular vesicles, irradiated tumor cell‐derived microparticles, IL‐1β, neutrophil, radiotherapy

## Abstract

Radiotherapy is known to trigger immunogenic cell death and activate local anti‐tumor immune responses. However, its systemic immunomodulatory effects remain poorly understood. Here, we discovered that irradiated tumor cell‐derived microparticles (RT‐MPs) are released into the circulation and subsequently taken up by neutrophils in the spleen. The mitochondrial DNA contained within RT‐MPs promotes the hyperactivation of neutrophils, leading to the secretion of interleukin‐1beta (IL‐1β) via the STING/NLRP3/GSDMD axis. IL‐1β, in turn, enhances the antigen‐presenting capacity of dendritic cells (DCs), which facilitates the formation of cytotoxic T lymphocytes (CTLs) in the spleen. These CTLs then contribute to the destruction of distant, non‐irradiated tumors. Our findings provide valuable insights into the mechanisms by which radiotherapy can directly modulate systemic anti‐tumor immunity, highlighting the potential for leveraging these effects to improve the efficacy of cancer treatment.

## Graphical abstract

Radiotherapy induces tumor cells to release microparticles (RT‐MPs) into the circulation. The mitochondrial DNA carried by these RT‐MPs activates the STING/NLRP3/GSDMD axis in splenic neutrophils, triggering IL‐1β secretion. This, in turn, enhances dendritic cell function and facilitates the formation of cytotoxic T lymphocytes, thereby promoting systemic antitumor immunity and ultimately driving the regression of distant tumors.

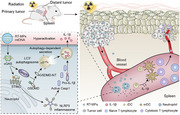

## 1 Introduction

Radiotherapy, a cornerstone therapeutic modality in oncology, is administered to over 50% of cancer patients with either curative intent or for palliative purposes [1, 2]. Its principal mechanism involves inducing DNA damage and reactive oxygen species (ROS) generation, ultimately leading to cancer cell death within the irradiated field [3, 4]. Notably, radiotherapy‐induced cell death exhibits immunogenic properties that can elicit systemic immune responses capable of reducing tumor burden at distant, non‐irradiated sites, a phenomenon termed the abscopal effect [5, 6]. While patients manifesting this effect demonstrate improved clinical outcomes, the abscopal response remains infrequent in clinical practice [7, 8], with its underlying mechanisms remaining incompletely understood. Elucidating the immunological mechanisms underlying radiotherapy‐mediated systemic responses could enhance therapeutic efficacy and improve patient survival.

Emerging evidence suggests the abscopal effect is associated with radiotherapy‐induced “in situ vaccination” [9]. This process involves immunogenic cell death of irradiated tumor cells, resulting in the release of double‐stranded DNA (dsDNA), damage‐associated molecular patterns (DAMPs), and tumor‐associated antigens (TAAs) [10, 11]. The dsDNA activates the cyclic GMP‐AMP synthase (cGAS)‐stimulator of interferon genes (STING) pathway, triggering type I interferon (IFN) production that promotes dendritic cells (DCs) activation [12]. Concurrently, DAMPs—including surface‐exposed calreticulin, released high mobility group protein B1 (HMGB1), and secreted adenosine triphosphate (ATP) – collectively facilitate DC recruitment, maturation, and antigen‐presenting capacity. Subsequently, DCs internalize TAAs and migrate to draining lymph nodes to prime tumor‐specific cytotoxic T lymphocytes (CTLs). These activated CTLs not only infiltrate irradiated tumor sites to eliminate residual cancer cells but also systemically circulate to eradicate distant metastases, thereby mediating cancer regression [13, 14]. While these findings illustrate radiotherapy's capacity to remodel the local tumor immune microenvironment, whether localized radiation can directly potentiate systemic anti‐tumor immunity remains uncertain.

Extracellular vesicles (EVs), comprising apoptotic bodies (ABs), microparticles (MPs), and exosomes (EXOs), serve as crucial mediators of intercellular communication [15, 16]. Recent studies reveal that radiotherapy significantly modifies EVs secretion profiles from tumor cells, with divergent biological outcomes reported [17, 18]. Our previous work demonstrated that irradiated tumor cell‐derived microparticles (RT‐MPs) exhibit direct tumoricidal effects and promote anti‐tumor immunity by polarizing macrophages toward the M1 phenotype [19]. Paradoxically, other investigation has shown that MPs from irradiated breast cancer cells carry programmed death‐ligand 1 (PD‐L1) and facilitate immune evasion [20]. These contradictory findings highlight the need for comprehensive characterization of radiation‐induced EVs subtypes and their cargo, as well as clarification of their context‐dependent roles in tumor progression and immune modulation.

In this investigation, we identified RT‐MPs as the predominant EV subtype capable of systemically enhancing anti‐tumor immunity and suppressing distant tumor growth among the three EV categories analyzed. Crucially, splenic involvement emerged as a prerequisite for this systemic immune activation. Mechanistic studies revealed that mitochondrial DNA (mtDNA) encapsulated within RT‐MPs drives neutrophil hyperactivation in the splenic compartment via coordinated activation of the STING/NLRP3/GSDMD signaling axis, culminating in robust interleukin‐1β (IL‐1β) secretion. This pro‐inflammatory cytokine subsequently potentiates DC maturation and antigen‐presenting functions, fostering the generation of CTLs. The systemically mobilized CTLs ultimately infiltrate non‐irradiated tumor sites, mediating abscopal tumor regression. Our findings delineate a novel spleen‐centered paradigm for radiotherapy‐induced systemic immunity, elucidating how localized radiation triggers mtDNA‐dependent innate immune activation to orchestrate adaptive anti‐tumor responses. This work advances our understanding of the abscopal effect by identifying RT‐MPs as key mediators of radiation‐induced systemic immune reprogramming and provides mechanistic insights for optimizing combinational radio‐immunotherapeutic strategies.

## 2 Results

### 2.1 RT‐MPs Inhibit Distant Tumor Growth Through Specific Immune Response

**FIGURE 1:**
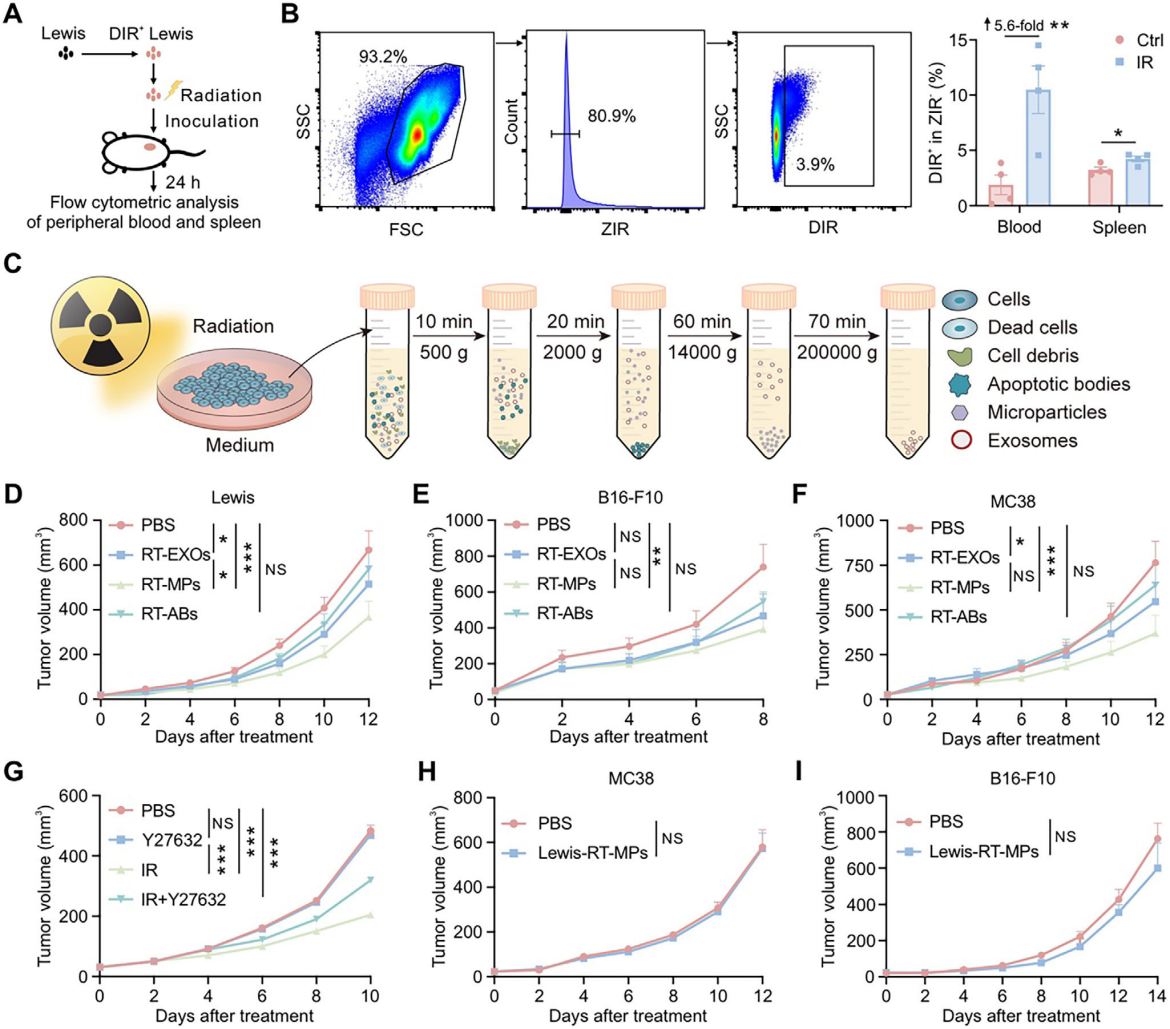
RT‐MPs suppress distant tumor growth via antigen‐specific immune response. (A) Flowchart illustrating the protocol for quantifying DIR^+^ viable cell populations. (B) Flow cytometry analysis of DIR^+^ viable cell percentages in blood and splenic tissues (*n* = 4). (C) Schematic diagram outlining the isolation workflow of RT‐EVs. (D) Tumor growth curves for the Lewis cell subcutaneous transplant model in the corresponding treatment groups (*n* = 7). (E) Tumor volume progression in B16‐F10 melanoma subcutaneous models following RT‐EV administration (*n* = 6–7). (F) Therapeutic response evaluation in MC38 colorectal adenocarcinoma xenografts treated with distinct RT‐EV formulations (*n* = 8). (G) Growth of right‐flank Lewis tumors (distant) following treatment with radiotherapy (IR) and the vesicle release inhibitor Y27632 (*n* = 8). (H,I) Therapeutic efficacy assessment of Lewis‐RT‐MPs in murine tumor models: MC38 colorectal adenocarcinoma (H, *n* = 9) and B16‐F10 melanoma (I, *n* = 6). *p < 0.05, **p < 0.01, and ***p < 0.001. Data are presented as mean ± SEM; two‐tailed unpaired *t*‐test for (B); two‐way ANOVA for (D–I).

To investigate the functional role of EVs in radiotherapy‐mediated systemic effects, we first evaluated radiation‐induced EV dynamics in vivo. Leveraging the lipid bilayer structure of EVs, we pre‐labeled murine Lewis lung carcinoma cell membranes with the lipophilic fluorescent dye DIR prior to delivering a 20 Gy radiation dose (Figure 1A). These labeled, irradiated cells were then subcutaneously implanted into the right flank of mice. Subsequent flow cytometric analysis 24 h post‐treatment revealed a significant elevation of DIR^+^ viable cells in both circulating blood and splenic tissues of irradiated groups compared to non‐irradiated controls (Figure 1B). Complementary near‐infrared fluorescence imaging confirmed enhanced DIR signal intensity within splenic regions in irradiation group (Figure S1), collectively suggesting radiotherapy promotes tumor‐derived EV (RT‐EV) generation and systemic dissemination. Guided by these observations, we hypothesized RT‐EVs might function as systemic immune modulators post‐radiation. We systematically isolated three EV subtypes from irradiated cell supernatants using differential centrifugation (Figure 1C). In accordance with MISEV2023 guidelines [21], transmission electron microscopy (TEM) and nanoparticle tracking analysis (NTA) were performed. The results validated the successful separation of these EV populations based on their distinct morphological and size characteristics (Figure S2A,B). Western blot analysis further validated the presence of canonical EV markers, including ARF6, KIF23, CD63, CD9, and TSG101 in the RT‐EV isolates (Figure S2C). Furthermore, treatment with the pan‐caspase inhibitor Z‐VAD‐FMK, used to suppress apoptosis, specifically reduced the yield of RT‐ABs but had no significant effect on the production of RT‐MPs (Figure S2D).

To functionally characterize RT‐EVs, we administered isolated EV subtypes intravenously to mice bearing subcutaneous xenografts four times at 2‐day intervals. Strikingly, RT‐MPs demonstrated potent anti‐tumor efficacy across multiple syngeneic models, significantly suppressing the growth of Lewis lung carcinomas (Figure 1D), B16‐F10 melanomas (Figure 1E), and MC38 colorectal tumors (Figure 1F).  While extended dosing intervals also inhibited tumor growth, the efficacy was reduced compared to the standard schedule (Figure S2E). In addition to intravenous administration, direct intratumoral injection of RT‐MPs also exhibited significant anti‐tumor activity (Figure S2F). To investigate the role of endogenous RT‐MPs in vivo, we inhibited their release using the ROCK1 inhibitor Y27632. This inhibition abolished the abscopal effect of radiotherapy on distant tumors (Figure 1G). Crucially, this effect exhibited tumor‐type specificity: RT‐MPs derived from Lewis cells (Lewis‐RT‐MPs) failed to inhibit the growth of antigenically distinct MC38 or B16‐F10 tumors (Figure 1H,I). These findings establish RT‐MPs as radiation‐induced mediators of tumor‐specific immune responses capable of eliciting systemic anti‐tumor effects against homologous malignancies.

### 2.2 The Spleen Contributes to Systemic Cancer Suppression Through RT‐MP‐Mediated Mechanisms

**FIGURE 2:**
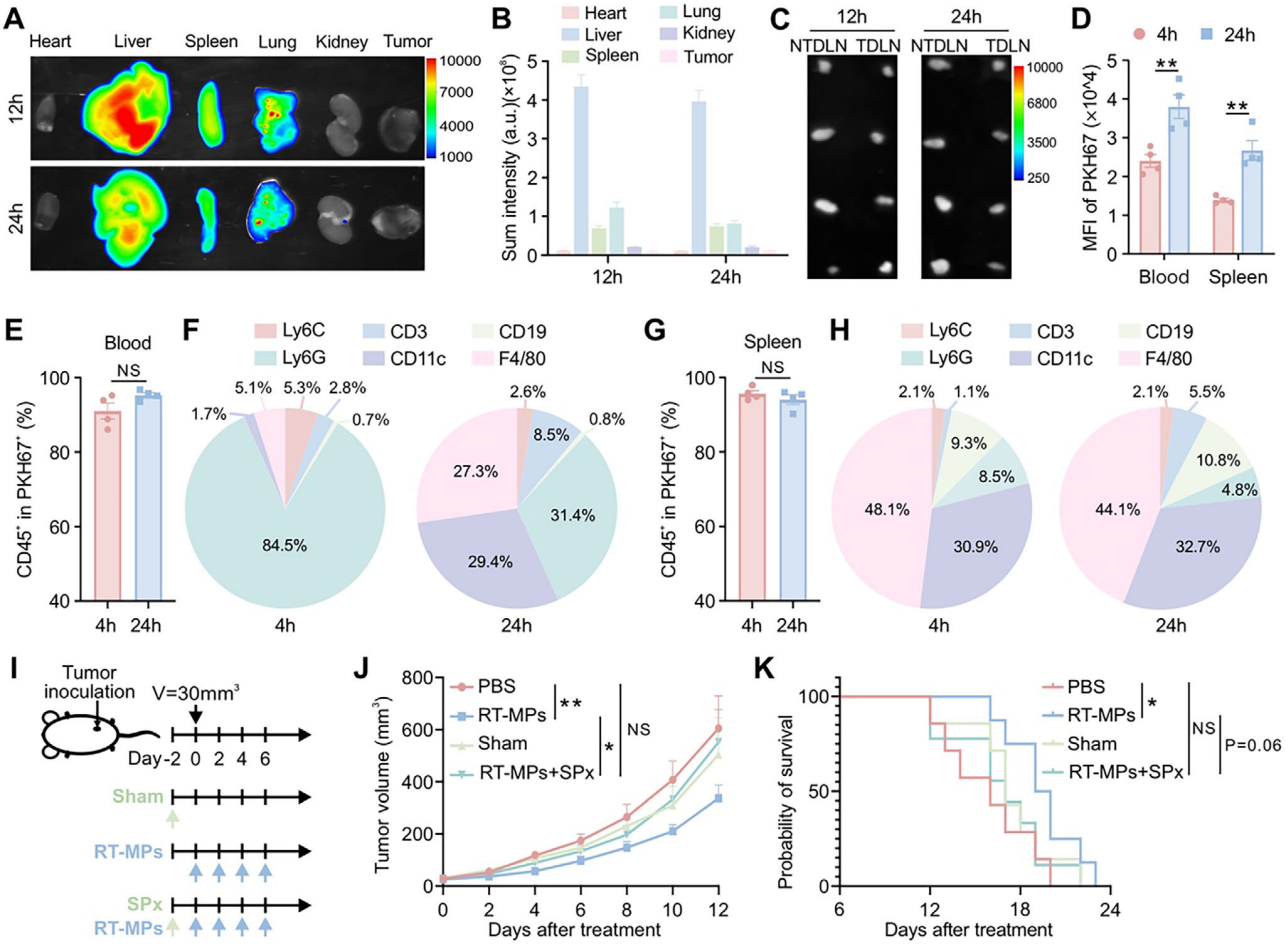
RT‐MPs are taken up by immune cells and accumulate in spleen to suppress tumor growth. (A,B) Representative near‐infrared fluorescence imaging of DIR signals in visceral organs and tumors (*n* = 5). (C) Near‐infrared fluorescence imaging of DIR signals in lymph nodes (*n* = 4). (D) Flow cytometric analysis of PKH67 mean fluorescence intensity (MFI) in live cells from the indicated groups (*n* = 4). (E) Proportion of CD45^+^ leukocyte within PKH67^+^ cells in blood (*n* = 4). (F) RT‐MP distribution patterns in peripheral blood leukocyte populations (*n* = 4). (G) Proportion of CD45^+^ leukocyte within PKH67^+^ cells in spleen (*n* = 4). (H) Quantitative profiling of RT‐MP cellular tropism in splenic leukocyte populations (*n* = 4). (I) Schematic illustration of the treatment plan. (J) Tumor growth curves of Lewis cell subcutaneous transplant model in corresponding treatment groups (*n* = 7–9). (K) Kaplan–Meier survival plot of Lewis‐bearing mice in corresponding groups described in (I). *p < 0.05, **p < 0.01, and ***p < 0.001. Data are presented as mean ± SEM; two‐tailed unpaired *t*‐test for (D, E, G); two‐way ANOVA for (J); log rank (Mantel‐Cox) test for (K).

To delineate the mechanistic basis of RT‐MP‐induced distant tumor control, we first mapped their systemic biodistribution in vivo. DIR‐labeled RT‐MPs were intravenously administered to Lewis tumor‐bearing mice, with organs harvested at 12 h and 24 h post‐injection for fluorescence quantification. Biodistribution analysis revealed predominant RT‐MP accumulation in hepatic tissue, followed by pulmonary and splenic compartments (Figure 2A,B). Notably, minimal DIR signal was detected in tumor masses, right inguinal lymph nodes (tumor‐draining lymph nodes, TDLN) and left inguinal lymph nodes (non‐tumor‐draining lymph nodes, NTDLN) (Figure 2A–C), suggesting RT‐MPs exert antitumor effects indirectly through immune modulation rather than directly killing cancer cells as we previously reported [19].

Then, we detected the cellular internalization of PKH67‐labeled RT‐MPs in mice after intravenous administration. Flow cytometric analysis of peripheral blood and spleen revealed sustained uptake, with elevated PKH67 levels persisting for over 24 h (Figure 2D). Further analysis indicated that over 90% of PKH67^+^ events localized to CD45^+^ leukocytes (Figure 2E). The uptake of RT‐MPs is nonspecific and is not restricted to a particular compartment, being evident in both the blood and the spleen (Figure 2F,H). Time‐resolved profiling revealed dynamic cellular uptake patterns: at 4 h post‐injection, neutrophils (Ly6G^+^, 84.5%) dominated RT‐MP internalization, with minor uptake by monocytes (Ly6C^high^, 5.3%) and macrophages (F4/80^+^, 5.1%), while lymphocytes (CD3^+^ and CD19^+^) and DCs (CD11c^+^) showed negligible uptake (Figure 2F). By 24 h, a pronounced shift occurred with DCs (29.4%) and macrophages (27.3%), concomitant with reduced neutrophil engagement (31.4%) (Figure 2F). Splenic analysis revealed distinct cellular handling: macrophages and DCs constituted the primary RT‐MP‐engaging populations, with stable uptake patterns between 4 and 24 h – contrasting sharply with the temporal dynamics observed in circulation (Figure 2G,H).

Given the spleen's centrality in systemic immunity, we performed splenectomy (SPx) prior to RT‐MP administration (Figure 2I). Strikingly, splenectomized mice completely lost RT‐MP‐induced tumor growth inhibition compared to intact counterparts (Figure 2J,K), unequivocally establishing the spleen as the critical hub for initiating RT‐MP‐mediated antitumor immunity.

### 2.3 RT‐MPs Elicit a Systemic Anti‐Tumor Effect by Engaging CD8^+^ T Cells, DCs, and Neutrophils

**FIGURE 3:**
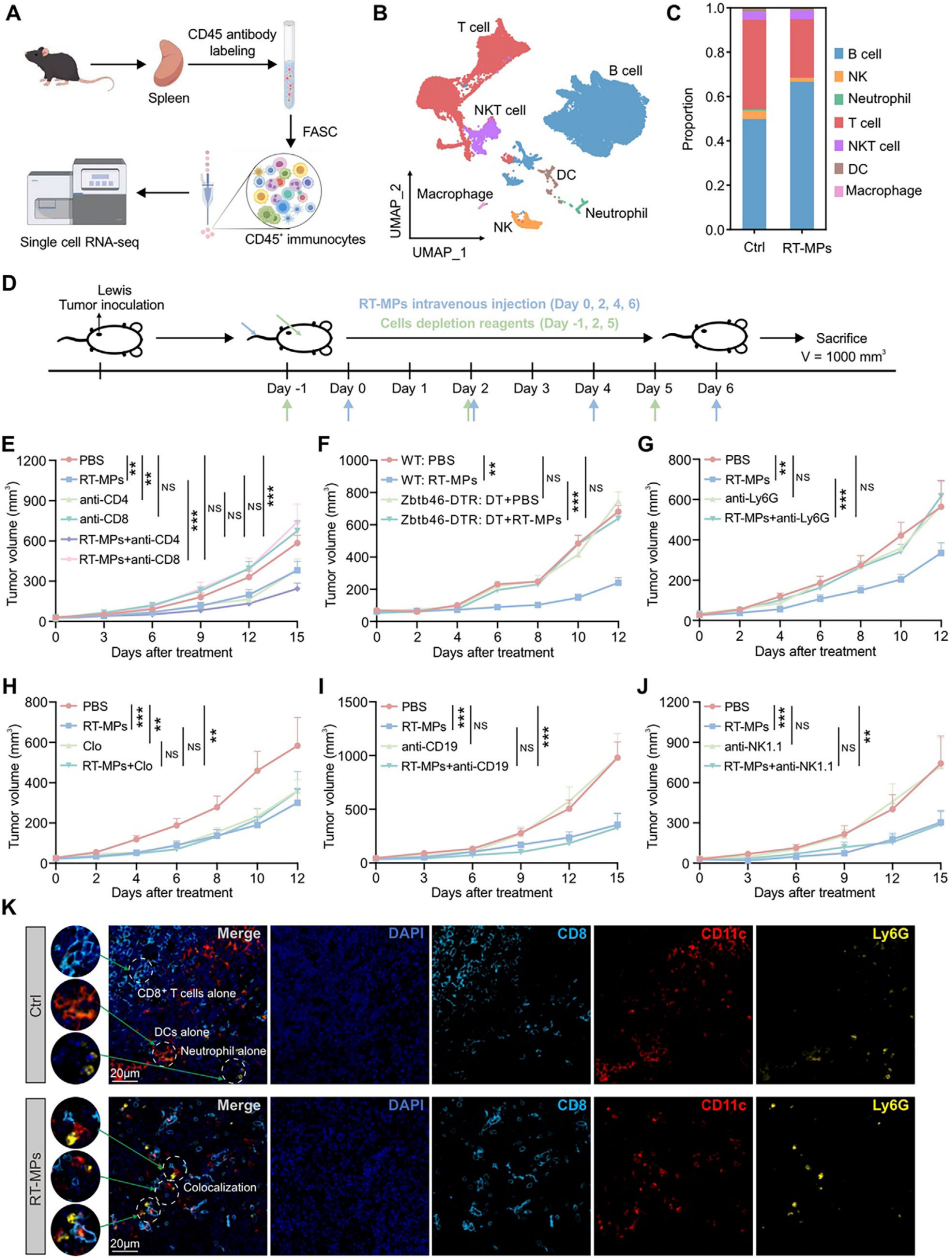
RT‐MPs inhibit distant tumor growth through DCs, neutrophils, and CD8^+^ T cells. (A) Outline of the immune cell isolation in spleen and single‐cell RNA‐seq analysis, made by Figdraw. (B) UMAP plot of all cells passed quality control from all samples, colored by cell subsets. (C) Histogram of single sample cell compositions. (D) Schematic illustration of the treatment plan. (E) Tumor growth curve of Lewis‐bearing C57BL/6 mice that were treated with RT‐MPs, concomitant with anti‐CD8 antibodies or anti‐CD4 antibodies (*n* = 9). (F) Tumor growth curve of Lewis‐bearing C57BL/6 mice and Lewis‐bearing Zbtb46‐DTR mice that were treated with RT‐MPs, concomitant with DT (*n* = 6–8). (G–J) Tumor growth curve of Lewis‐bearing C57BL/6 mice that were treated with RT‐MPs, concomitant with anti‐Ly6G antibodies (G, *n* = 7–10), clodronate liposomes (H, *n* = 5–7), anti‐CD19 antibodies (I, *n* = 7–8), and anti‐NK1.1 antibodies (J, *n* = 6–9). (K) Representative immunofluorescence staining of CD8^+^ T cells (cyan), DCs (red), and neutrophils (yellow) in the spleen. Scale bar, 20 µm. *p < 0.05, **p < 0.01, and ***p < 0.001. Data are presented as mean ± SEM; two‐way ANOVA for (E–J).

To delineate the immunomodulatory landscape of RT‐MPs within splenic compartments, we conducted single‐cell RNA sequencing (scRNA‐seq) on CD45^+^ immune cells from treated spleens (Figure 3A). Unsupervised clustering identified seven major immune populations: B cells, T cells, natural killer (NK) cells, NKT cells, neutrophils, DCs, and macrophages (Figure 3B and Figure S3A). Quantitative analysis revealed RT‐MP treatment induced significant lymphoid remodeling, characterized by B cell expansion coupled with contraction of T cells and NK cells (Figure 3B,C).

To functionally identify effector cell types, we employed a combinatorial depletion strategy targeting key immune subsets prior to RT‐MP administration (Figure 3D). Flow cytometric validation confirmed efficient depletion of CD4^+^ T cells (anti‐CD4), CD8^+^ T cells (anti‐CD8), DCs (diphtheria toxin [DT]), neutrophils (anti‐Ly6G), macrophages (clodronate liposomes), B cells (anti‐CD19), and NK cells (anti‐NK1.1) (Figure S3B–H). Strikingly, therapeutic efficacy was completely abrogated upon depletion of CD8^+^ T cells, DCs, or neutrophils (Figure 3E–G), while targeting CD4^+^ T cells, macrophages, B cells, or NK cells preserved RT‐MP‐mediated tumor suppression (Figure 3H–J).

Ligand‐receptor pairing analysis further revealed enhanced interaction networks between neutrophils, DCs, and T cells in RT‐MP‐treated spleens (Figure S3I), This finding implies a high level of coordinated cross‐talk between these cellular populations, which was further confirmed by multi‐color immunofluorescence staining (Figure 3K). These multimodal data establish a tripartite immune axis wherein RT‐MPs engage splenic neutrophils and DCs to license CD8^+^ T cell‐mediated tumor control.

### 2.4 Systemic T Cell Activation Underpins RT‐MP‐Mediated Antitumor Immunity

**FIGURE 4:**
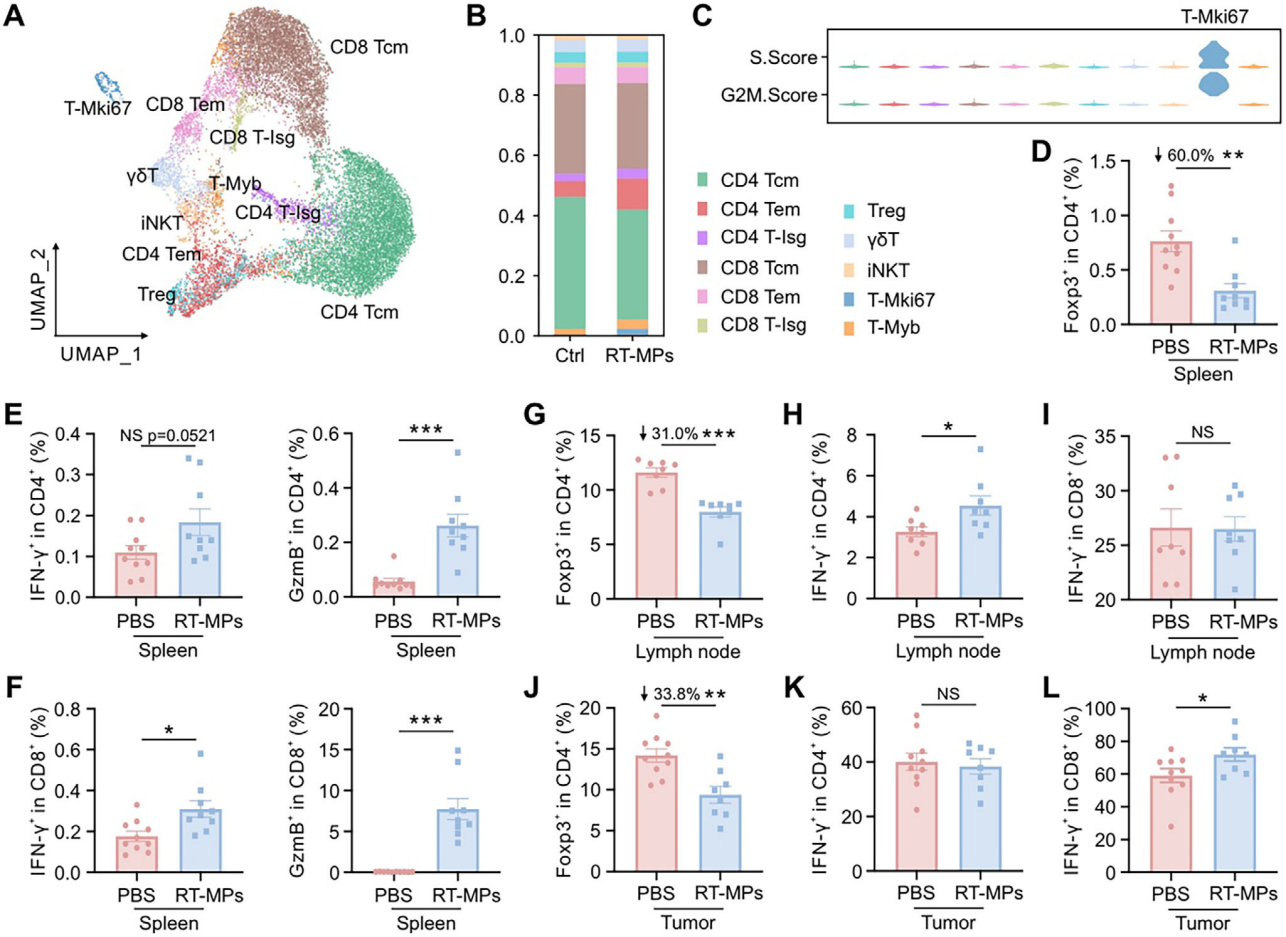
RT‐MPs systematically activate T cell‐mediated anti‐tumor immunity. (A) UMAP plot of T cell from all samples, colored by identified cell clusters. (B) Stacked bar plot showing the proportion of T cell clusters. (C) Violin plot of S.Score and G2/M.Score in each T cell cluster. (D–F) Flow cytometry analysis of each T cell subset in the spleen (*n* = 9–10). Frequency of Tregs (Foxp3^+^) (D) and Th1 (IFN‐γ^+^ or GzmB^+^) (E) in CD4^+^ T cells, CTLs (IFN‐γ^+^ or GzmB^+^) (F) within CD8^+^ T cells. (G–I) Flow cytometry analysis of each T cell subset in the lymph node (*n* = 8). Frequency of Tregs (G) and Th1 (H) in CD4^+^ T cells. CTL frequency in CD8^+^ T cells (I). (J–L) Flow cytometry analysis of each T cell subset in the tumor (*n* = 8–10). Frequency of Tregs (J) and Th1 (K) in CD4^+^ T cells. CTL frequency in CD8^+^ T cells (L). *p < 0.05, **p < 0.01, and ***p < 0.001. Data are presented as mean ± SEM; two‐tailed unpaired *t*‐test for (D–L).

To resolve the splenic T cell dynamics induced by RT‐MPs, we performed high‐resolution T cell subtyping, categorizing splenocytes into 11 functionally distinct subset [22, 23, 24, 25, 26], including CD4‐central memory T cell (Tcm), CD4‐effector memory T cell (Tem), CD4‐interferon‐stimulated genes (Isg), regulatory T cells (Tregs), CD8‐Tcm, CD8‐Tem, CD8‐Isg, γδ T cells, invariant natural killer T cells (iNKT), T‐Mki67, and T‐Myb (Figure 4A and Figure S4A). Compositional analysis revealed memory T cells dominated the splenic T cell landscape in both groups, with RT‐MP treatment inducing a marked expansion of the proliferative T‐Mki67^+^ subset (Figure 4B,C). Single‐cell transcriptomics further demonstrated significant downregulation of *Tgfβ1* – a potent immunosuppressive cytokine and Treg inducer [27] – in RT‐MP‐exposed T cells (Figure S4B,C), suggesting dual mechanisms of enhanced T cell activation and reduced immunosuppression.

To comprehensively map systemic immunomodulatory effects, we administered RT‐MPs (q2d ×4 doses) to Lewis tumor‐bearing mice and performed multiparametric flow cytometric analysis across three key immunological compartments, revealing distinct compartment‐specific immunomodulatory effects (Figure S4D). Notably, the splenic analysis demonstrated marked reductions in Tregs (Figure 4D), concomitant with significant expansion of both cytotoxic CD4^+^GzmB^+^ Th1 cells exhibiting enhanced effector function (Figure 4E) and amplified CTL populations (Figure 4F). In tumor‐draining lymph nodes, we observed parallel reductions in Treg frequencies (Figure 4G) coupled with enhanced Th1 polarization (Figure 4H), though CTL proportions remained comparable to controls (Figure 4I). Most critically within the tumor microenvironment, RT‐MP treatment mediated substantial reductions in Treg infiltration (Figure 4J) alongside robust CTL accumulation (Figure 4L), despite maintaining baseline Th1 cell levels (Figure 4K). Collectively, these findings demonstrate that RT‐MPs systemically enhance antitumor immunity through splenic immune reprogramming, resulting in increased CTL/Th1 cell infiltration and reduced Treg accumulation in both peripheral and tumor microenvironments.

### 2.5 RT‐MPs Potentiate Splenic DC Activation and Antigen Presentation

**FIGURE 5:**
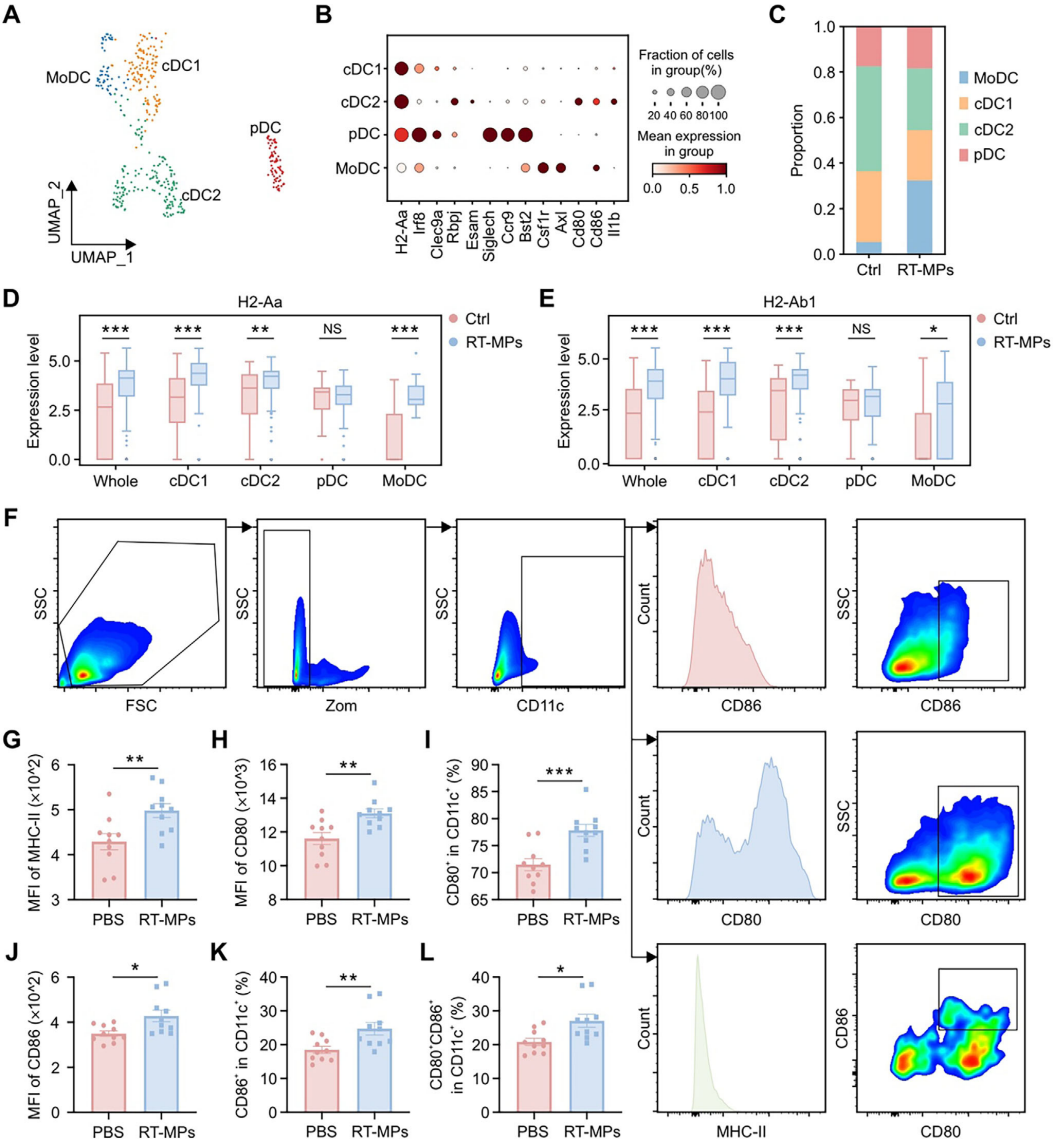
RT‐MPs boost splenic DC activation and antigen presentation. (A) UMAP plot of DCs from spleen of PBS‐treated and RT‐MP‐treated mice, colored by identified cell clusters. (B) Bubble heatmap showing selected genes in DC clusters. (C) Stacked bar plot showing the proportion of DC clusters originating from different samples. (D) Comparison of *H2‐Aa* expression in each cluster of cells. (E) Comparison of *H2‐Ab1* expression in each cluster of cells. (F) Flow cytometry gating strategy for the detection of DCs in the spleen. (G) Flow cytometry analysis of the MHC‐II expression on DCs in corresponding treatment groups (*n* = 10). (H,I) Flow cytometry analysis of the CD80 expression (H) and frequency of CD80^+^ (I) in CD11c^+^ DCs (*n* = 10). (J,K) Flow cytometry analysis of the CD86 expression (J) and frequency of CD86^+^ (K) within CD11c^+^ DCs (*n* = 10). (L) Frequency of CD80^+^CD86^+^ co‐expression in CD11c^+^ DCs (*n* = 10). *p < 0.05, **p < 0.01, and ***p < 0.001. Data are presented as mean ± SEM; two‐tailed unpaired *t*‐test for (D, E, G–L).

To delineate DC subset‐specific responses to RT‐MP treatment, we performed high‐dimensional clustering of splenic DCs, resolving four functionally distinct populations: conventional DC1 (cDC1), cDC2, plasmacytoid DCs (pDCs), and monocyte‐derived DCs (MoDCs) (Figure 5A,B). While total DC frequencies remained unchanged (Figure 3C), subset redistribution occurred with MoDCs – mediators of CD8^+^ T cell cross‐priming [28] – expanding significantly at the expense of cDC2 populations (Figure 5C). Differential gene expression analysis revealed pronounced upregulation of antigen presentation machinery components, particularly MHC class II α‐chain (*H2‐Aa*) and β‐chain (*H2‐Ab1*) genes across cDC1, cDC2, and MoDC subsets (Figure 5D,E and Figure S5A). Functional enrichment analyses (GO/KEGG) further confirmed preferential activation of antigen processing/presentation pathways in RT‐MP‐treated DCs (Figure S5B). Flow cytometric validation demonstrated elevated MHC‐II surface expression (Figure 5G) and co‐stimulatory activation, evidenced by increased CD80^+^ (Figure 5H,I), CD86^+^ (Figure 5J,K), and dual CD80^+^CD86^+^ (Figure 5L) DC populations. These molecular and functional adaptations collectively establish RT‐MPs as potent activators of DC licensing through three interconnected mechanisms: first, the preferential expansion of MoDCs exhibiting enhanced cross‐priming capacity; second, the reinforcement of antigen‐presenting machinery via elevated MHC‐II biosynthesis and complex assembly; third, the acquisition of co‐stimulatory proficiency through amplified surface expression of CD80 and CD86. This coordinated triad of DC activation – spanning cellular subset specialization, antigen‐presentation optimization, and co‐stimulatory signal amplification – functionally integrates RT‐MP‐mediated innate immune stimulation with the establishment of tumor‐targeted adaptive immunity, thereby mechanistically linking DC activation to systemic antitumor responses.

### 2.6 mtDNA Within RT‐MPs Triggers STING/NLRP3/GSDMD‐Mediated IL‐1β Production in Neutrophils

**FIGURE 6:**
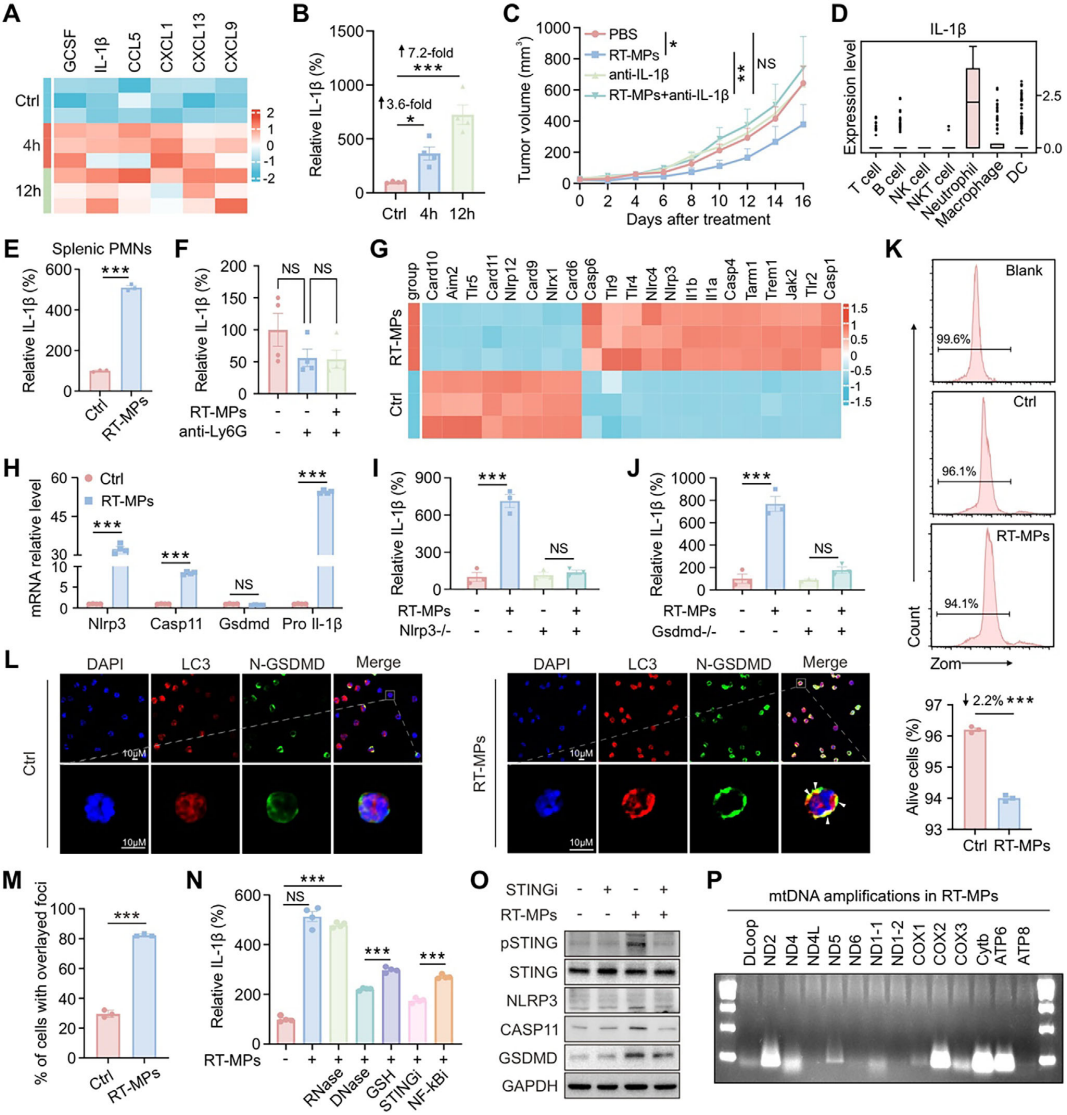
RT‐MPs trigger neutrophil IL‐1β release via the STING/NLRP3/GSDMD axis in the spleen. (A) Heatmap of cytokine array results in the spleen after treatment in corresponding treatment groups (*n* = 3). (B) ELISA analysis of IL‐1β content in the spleen after treatment of RT‐MPs (*n* = 4). (C) Tumor growth curves of Lewis cell subcutaneous transplant model in corresponding treatment groups (*n* = 6). (D) Comparison of *Il‐1β* expression in each cluster of cells. (E) Quantification of RT‐MP‐induced IL‐1β secretion in splenic neutrophil culture supernatants by standardized ELISA (*n* = 3). (F) ELISA analysis of IL‐1β content in the spleen after treatment in corresponding treatment groups (*n* = 4). (G) Heatmap of genes analyzed by RNA‐seq on neutrophils treated with RT‐MPs (*n* = 3). (H) qRT‐PCR quantification of RT‐MP‐induced transcripts in neutrophils (*n* = 4). (I) ELISA analysis of IL‐1β secretion levels in neutrophil supernatants from WT and *Nlrp3^−/−^* mice following RT‐MP treatment (*n* = 3). (J) ELISA analysis of IL‐1β secretion levels in neutrophil supernatants from WT and *Gsdmd^−/−^* mice following RT‐MP treatment (*n* = 3). (K) Flow cytometry assessment of neutrophil viability rates following RT‐MP treatment (*n* = 3). (L) Representative immunofluorescence images of RT‐MP‐treated neutrophils co‐stained for N‐GSDMD (green) and LC3 (red). Scale bar, 10 µm. (M) The percentage of N‐GSDMD and LC3 double‐positive cells was quantified (*n* = 3). (N) ELISA analysis of IL‐1β content in the supernatant of neutrophils after treatment in corresponding treatment groups (*n* = 4). (O) Western blots of pSTING, STING, NLRP3, CASP11, GSDMD, and GAPDH expression in neutrophils treated with RT‐MPs or STING inhibitors (*n* = 3). (P) RT‐PCR of mitochondrial DNA in RT‐MPs (*n* = 3). *p < 0.05, **p < 0.01, and ***p < 0.001. Data are presented as mean ± SEM; two‐tailed unpaired t‐test for (E, H, K, M); one‐way ANOVA for (B, F, I, J, N); two‐way ANOVA for (C).

Building on the spleen's central role in RT‐MP‐mediated immunity, we then mapped cytokine/chemokine dynamics post‐treatment using multiplex cytokine profiling. RT‐MP administration induced significant splenic upregulation of pro‐inflammatory mediators, including granulocyte colony‐stimulating factor (GCSF), interleukin‐1beta (IL‐1β), CC‐chemokine ligand 5 (CCL5), CXC‐chemokine ligand 1 (CXCL1), CXCL13, and CXCL19 (Figure 6A and Figure S6A). Given IL‐1β’s pivotal role in DC maturation [29], we focused on its spatiotemporal regulation. The standard curve for detecting IL‐1β is shown in Figure S6B. ELISA quantification revealed a time‐dependent IL‐1β elevation in splenic tissue (Figure 6B). Moreover, suppression of RT‐MP release blocked the ability of irradiation to elevate splenic IL‐1β (Figure S6C). Anti‐IL‐1β neutralization completely abrogated RT‐MP‐induced tumor suppression (Figure 6C), establishing its non‐redundant role.

Subsequently, we aimed to further explore the cellular source of IL‐1β. As shown in Figure 6D, single‐cell transcriptomics demonstrated that IL‐1β transcription was broadly induced in splenic immune cells like macrophages and DCs after RT‐MP treatment, but was most pronounced in neutrophils. In vitro validation using splenic neutrophils confirmed RT‐MPs directly stimulate neutrophil IL‐1β secretion (Figure 6E), as well as from peripheral blood neutrophils (PBNEs; Figure S6D) and bone marrow neutrophils (BMNEs; Figure S6E). In comparison, RT‐MPs induced only modest IL‐1β secretion from bone marrow‐derived macrophages (BMDMs), which was substantially lower than that from neutrophils (Figure S6F), and failed to elevate IL‐1β levels in bone marrow‐derived dendritic cells (BMDCs; Figure S6G). Analysis of immune cell composition indicated that the splenic neutrophil frequency remained stable (Figure S6H). To further characterize the functional changes induced by RT‐MPs, splenic Ly6G^+^ cells were sorted from Lewis‐tumor‐bearing mice. The results demonstrated that RT‐MP treatment promoted an N1‐like pro‐inflammatory phenotype in these cells, without inducing a canonical feature of myeloid‐derived suppressor cells (MDSCs) (Figure S7). Critically, neutrophil depletion with anti‐Ly6G antibodies in vivo abolished splenic IL‐1β induction (Figure 6F), confirming their role as the principal IL‐1β source.

To explore the mechanism of RT‐MP‐induced IL‐1β secretion, we performed RNA‐seq on RT‐MP‐treated neutrophils. The different gene expression levels between the control group and the RT‐MP group are shown in Figure S6I. The KEGG analysis revealed significant enrichment of the NOD‐like receptor (NLR) signaling pathway (Figure S6J), which was previously reported to promote the production of IL‐1β [30]. Therefore, the differentially expressed genes related to the NLR signaling pathway were displayed in Figure 6G, with upregulated *Nlrp3*, *Casp11*, and *Pro Il‐1β* transcripts in RT‐MP‐treated neutrophils (Figure 6H). A similar, though less pronounced, upregulation was also observed in BMDMs (Figure S6K). Genetic ablation studies in *Nlrp3* ^−/−^ and *Gsdmd* ^−/−^ mice confirmed the NLRP3 inflammasome/GSDMD axis as essential for IL‐1β production (Figure 6I,J). Previous studies have established that IL‐1β can be released through pyroptosis or autophagy‐dependent mechanisms [31, 32]. To determine the pathway involved in RT‐MP‐induced secretion, we first measured LDH release in the culture media of treated neutrophils and observed no significant increase (Figure S6L), indicating the absence of pyroptosis. Flow cytometry confirmed that RT‐MP treatment did not alter the proportion of viable neutrophils (Figure 6K). The observed changes in neutrophil phagocytic capacity can be attributed to their naturally short half‐life (Figure S6M). Together, these data indicate that IL‐1β secretion occurs independently of pyroptotic cell death. Instead, immunofluorescence analysis revealed a notable increase in the percentage of neutrophils double‐positive for N‐GSDMD and LC3 following RT‐MP treatment (Figure 6L,M). Consistent with an autophagy‐dependent process, the secretion was blocked by geldanamycin (Gel) (Figure S6N), a known inhibitor that prevents cargo loading into autophagosomes and inhibits Hsp90‐mediated IL‐1β trafficking into these vesicles [33]. Furthermore, inhibiting autophagy with 3‐methyladenine (3‐MA) reduced IL‐1β release, whereas the pyroptosis inhibitor disulfiram had no such effect (Figure S6O,P). These results collectively suggest that RT‐MPs induce IL‐1β release through an autophagy‐dependent mechanism rather than via pyroptosis.

Studies have shown that inflammasomes can be activated by RNA, DNA, and ROS through the cGAS/STING pathway or NF‐κB pathway [34]. Based on this rationale, we employed RNase to degrade RNA, DNase to degrade DNA, L‐glutathione (GSH) to scavenge ROS, a STING inhibitor (STINGi) to block cGAS/STING pathway, and a NF‐κB inhibitor (NF‐κBi) to suppress NF‐κB pathway. We observed that RT‐MP‐induced IL‐1β secretion was modulated by DNase and STINGi (Figure 6N). Molecular validation through western blot confirmed that RT‐MPs elevated the protein expression of pSTING, NLRP3, CASP11, and GSDMD in neutrophils, with STINGi pretreatment effectively reversing these activation markers (Figure 6O). Genetic material characterization via RT‐PCR revealed that RT‐MPs contained mitochondrial DNA (mtDNA) (Figure 6P). These data collectively establish a mtDNA‐STING‐NLRP3‐GSDMD signaling axis as the central mechanism governing RT‐MP‐induced IL‐1β secretion in neutrophils, where radiation‐generated mtDNA cargo initiates sequential activation of cytosolic DNA sensing, inflammasome assembly, and pore‐forming protein processing to drive cytokine release.

### 2.7 IL‐1β Promotes CD8^+^ T Cell Responses Through Dendritic Cell Activation and Improved Antigen Presentation

**FIGURE 7:**
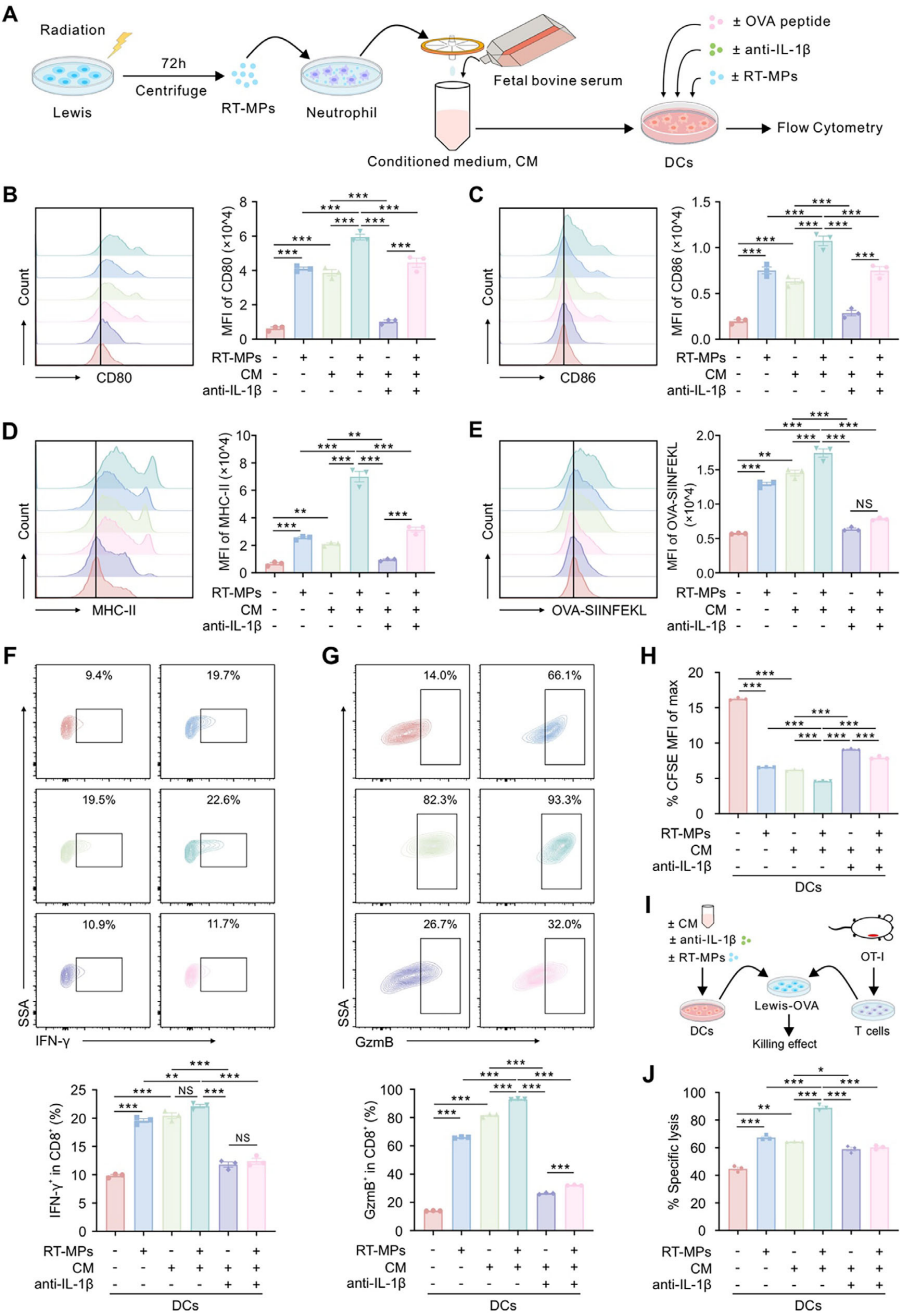
IL‐1β potentiates DC‐mediated antigen presentation and subsequent T cell activation. (A) Experimental workflow for detecting the profiling of CD80, CD86, MHC‐II and OVA‐SIINFEKL in DCs treated with RT‐MPs, CM, OVA peptide, and anti‐IL‐1β. (B‐E) The level of CD80 (B), CD86 (C), MHC‐II (D), and OVA‐SIINFEKL (E) in DCs in corresponding treatment groups analyzed by flow cytometry (*n* = 3). (F,G) The level of IFN‐γ (F) and GzmB (G) in CD8^+^ T cells in corresponding treatment groups analyzed by flow cytometry (*n* = 3). (H) The level of CFSE in CD8^+^ T cells in corresponding treatment groups analyzed by flow cytometry (*n* = 3). (I) Flowchart for detecting killing effect of OT‐I T cells to Lewis‐OVA. (J) Specific lysis of Lewis‐OVA tumor cells by OT‐I T cells pretreated with the indicated DCs in corresponding treatment groups (*n* = 3). *p < 0.05, **p < 0.01, and ***p < 0.001. Data are presented as mean ± SEM; one‐way ANOVA for (B–H, J).

Given IL‐1β’s established role in enhancing DCs antigen presentation [35, 36] and our prior demonstration of DC dependency in RT‐MP‐mediated immunity (Figure 3F), we hypothesized that neutrophil‐derived IL‐1β bridges innate and adaptive immunity through DC licensing. Consistent with this hypothesis, analysis of ligand‐receptor interactions from splenic scRNA‐seq data showed that RT‐MP treatment enhanced immunostimulatory while diminishing immunosuppressive crosstalk between DCs and T cells (Figure S5C). To formally test this, we collected conditioned media (CM) of RT‐MP‐treated neutrophils to culture bone marrow‐derived DCs in the presence/absence of OVA peptide and IL‐1β neutralizing antibody (Figure 7A). Multiparametric flow cytometric evaluation demonstrated three principal mechanistic insights: RT‐MP monotherapy or CM alone elevated DC co‐stimulatory markers, evidenced by significant increases in expression of CD80 and CD86, while combined RT‐MP+CM treatment produced synergistic enhancement of these activation markers (Figure 7B,C). Furthermore, CM exposure specifically amplified MHC‐II surface expression levels and doubled OVA‐SIINFEKL⁺ DC frequency, effects strictly mediated through IL‐1β‐dependent mechanisms (Figure 7D,E). Functional validation experiments confirmed complete IL‐1β dependency, with anti‐IL‐1β neutralizing antibodies effectively reversing CM‐induced DC maturation by achieving significant reduction in CD80, CD86, and MHC‐II expression profiles. In contrast, exogenous IL‐1β increased the expression of these markers in DCs (Figure S9A–C).

To assess downstream T cell consequences, we primed OT‐I CD8^+^ T cells (purity >95%, Figure S8A) with CM‐educated DCs and assessed their production of IFN‐γ and GzmB (Figure S8B). As shown in Figure 7F,G, CM‐primed DCs elicited robust T cell activation characterized by elevation in IFN‐γ‐producing cells and expansion of GzmB‐expressing cytotoxic effectors. Quantitative proliferation analysis through CFSE dilution showed that the proportion of CFSE^high^ T cells in CM‐treated DC group was significantly lower than that in control group (Figure 7H), indicating that CM could activate DCs and further increase the proliferation of CD8^+^ T cells. Moreover, functional validation using tumor cell lysis assays confirmed superior cytotoxic potential, showing increased specific killing of Lewis‐OVA target cells compared to baseline responses (Figure 7I,J), collectively establishing the functional competence of CM‐educated DCs in driving antigen‐specific CD8⁺ T cell responses. Crucially, IL‐1β neutralization reversed all CM‐DC effects, greatly reducing T cell activation, proliferation, and cytotoxicity (Figure 7F–J). Consistent with IL‐1β’s instructive role, DCs treated directly with recombinant IL‐1β similarly enhanced T cell effector functions (IFN‐γ, GzmB) and cytotoxicity (Figure S9D–F), and systemic IL‐1β administration inhibited tumor growth in vivo (Figure S9G,H). These data establish a sequential immunologic cascade: RT‐MP‐educated neutrophils release IL‐1β to license DCs for enhanced antigen presentation and co‐stimulation, which drive tumor‐specific CD8^+^ T cell expansion and effector differentiation.

## 3 Discussion

**FIGURE 8:**
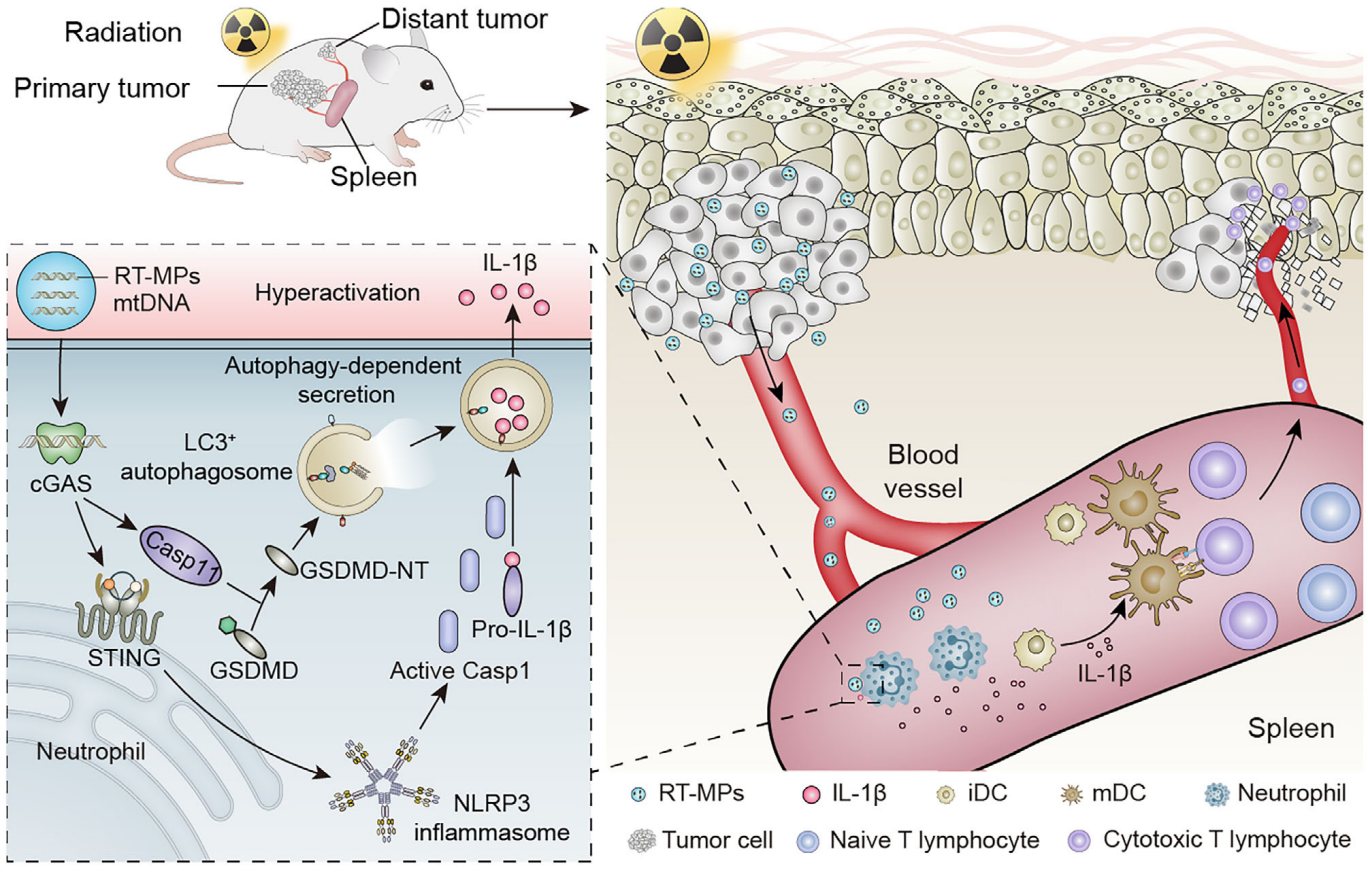
Schematic illustration shows the biological function and mechanism of RT‐MPs in radiotherapy‐induced systematic anti‐tumor immunity.

Radiotherapy has emerged as a potent inducer of immunogenic cell death, capable of eliciting the abscopal effect through anti‐tumor immunity [37]. While prior investigations predominantly focused on localized immune remodeling within irradiated tumor beds and TDLNs, the mechanisms governing systemic immune activation remain poorly defined. Our study unveils a novel spleen‐centric paradigm: Radiotherapy triggers tumor cells to release EVs, predominantly microparticles (RT‐MPs), which traffic to splenic compartments to orchestrate systemic anti‐tumor immunity. Crucially, RT‐MP‐mediated neutrophil activation via the mtDNA/STING/NLRP3/GSDMD axis drives IL‐1β‐dependent DC licensing, culminating in splenic CTL expansion and distant tumor regression. These findings provide valuable insights into how radiotherapy modulates the systemic anti‐tumor immunity and the molecular mechanisms underlying radiotherapy‐induced abscopal effect (Figure 8).

EVs, nanoscale carriers of bioactive cargo (proteins, nucleic acids, lipids), exhibit context‐dependent roles in vascular formation, tissue repair, immune response, and coagulation response [38, 39, 40]. Previous investigations have reported that tumor‐derived MPs under natural state could inhibit anti‐tumor immunity and promote angiogenesis through tumor microenvironmental modulation [41, 42]. Furthermore, emerging evidence reveals that hypoxic tumor cell‐derived MPs promote pulmonary metastasis via CCL2‐mediated macrophage recruitment [43]. Our previous work demonstrated that RT‐MPs paradoxically prevented lung metastasis through neutrophils and macrophages polarization [44], yet the specific bioactive components within RT‐MPs remained unidentified. The current study extends these observations, demonstrating RT‐MPs as master regulators of abscopal immunity across multiple syngeneic models in a neutrophil‐dependent but macrophage‐independent manner. The identification of mtDNA as the critical RT‐MP cargo activating STING/NLRP3 signaling resolves long‐standing questions about radiation‐induced EV bioactivity, positioning mtDNA as a novel DAMP in radiotherapy contexts.

Traditional paradigms of radiation immunity emphasize DC/T cells axis dominance [45, 46]. Other immune cells such as macrophages, and NK cells, are also involved in radiotherapy‐induced abscopal effect [17, 47, 48]. However, whether neutrophils are participated is not well understood. In this study, we find that neutrophils are indispensable intermediaries in systemic immune response activated by radiotherapy. Despite their controversial role in radioresistance (via neutrophil extracellular traps) and radiation toxicity [49, 50], we demonstrate neutrophils undergo functional reprogramming upon RT‐MP uptake, transitioning from transient first responders to sustained cytokine hubs. This aligns with emerging evidence of neutrophil plasticity, where microenvironmental cues dictate pro‐ vs anti‐tumor polarization [51]. The spatial specificity of splenic neutrophil activation further highlights organ‐specific immune education mechanisms warranting deeper exploration.

Studies have shown that cytokines such as IL‐2 and GM‐CSF can increase the incidence of radiotherapy‐induced abscopal effect, establishing cytokines as critical mediators of radiotherapy‐associated immune responses [52, 53]. Clinical observations in hepatocellular carcinoma patients demonstrating radiotherapy‐induced elevation of serum IL‐1β, IL‐4, and IL‐6 levels during abscopal response [54] prompted our mechanistic investigation into IL‐1β’s role in radiation‐triggered immunity. While IL‐1β exhibits context‐dependent duality in cancer biology – promoting tumor progression through cancer stem cell activation and pro‐tumorigenic myeloid differentiation [55, 56] versus suppressing metastasis via E‐cadherin downregulation in metastatic precursors [57] – our findings reveal its beneficial immunostimulatory role when mediated through radiation‐specific mechanisms. Clinically relevant correlations between elevated intratumoral IL‐1β and reduced hepatocellular carcinoma recurrence [58] suggest that its functional outcomes depend on concentration, spatial distribution, and target cell specificity. Mechanistically, we delineate a non‐canonical activation route: mtDNA‐STING priming licenses NLRP3 inflammasome assembly, driving IL‐1β secretion via GSDMD pore formation without pyroptosis – a lytic cell death pathway potentially detrimental to sustained cytokine production. This neutrophil‐derived IL‐1β surge propagates systemic immunity through DC activation and antigen presentation enhancement, culminating in expanded CD8⁺ T cell populations exhibiting amplified cytotoxic potential and tumoricidal activity. Our work establishes the spleen as a critical IL‐1β production hub during radiotherapy and elucidates a mtDNA‐STING‐IL‐1β axis that functionally connects radiation‐induced neutrophil activation to adaptive antitumor cytotoxicity, providing mechanistic rationale for IL‐1β’s beneficial role in radiation‐mediated immune activation.

Therapeutically, our findings reveal several exploitable mechanisms: circulating RT‐MP quantification could serve as predictive biomarkers for abscopal response, strategic STING pathway activation might synergize with radiotherapy to augment systemic immunity, and temporally controlled neutrophil manipulation could optimize treatment outcomes. Critical limitations require consideration, including current technical constraints in establishing definitive causal relationships between RT‐MPs and abscopal effects due to the inability of in vivo EV secretion blockade, the probable existence of functionally cooperative molecular cargo (e.g., oxidized lipids and non‐coding RNAs) alongside mtDNA requiring comprehensive compositional analysis, and the necessity to validate radiation parameters in human‐relevant models to assess clinical applicability.

In conclusion, this study repositions radiotherapy from a localized DNA‐damaging modality to a systemic immune primer via EV‐mediated interorgan communication. By mapping the RT‐MP/neutrophil/IL‐1β/DC/CTL axis, we provide a mechanistic scaffold for leveraging radiation‐induced EVs in combinatorial immunotherapy. Future efforts to engineer RT‐MP mimetics or optimize radiation schedules for enhanced EV production could transform radiotherapy into a systemic immune‐oncology tool.

## 4 Materials and Methods

### 4.1 Cell Lines and Cell Culture

Murine Lewis lung cancer cell (RRID: CVCL_S007), murine B16‐F10 melanoma cells (RRID: CVCL_0159), and murine colon cancer line MC38 (RRID: CVCL_B288) were purchased from the China Center for Type Culture Collection (Wuhan, China). The cell lines were routinely tested to confirm the absence of mycoplasma. The chicken ovalbumin (OVA) stably transfected cell line (Lewis‐OVA) was established by lentivirus transfection. HEK293T cells (RRID: CVCL_0063) were transfected with the vectors containing genes of OVA and packaged lentivirus vectors (psPAX2 and pMD2.G). After 48 h, the supernatants were harvested and filtered using a 0.45 µm filter. Lewis cells were then infected using the filtered supernatants with the addition of polybrene (10 µg/mL) (Sigma–Aldrich). Stably transfected cells were screened using puromycin (2 µg/mL) and then selected for monoclonal amplification. The final stably transfected monoclonal cell lines for experiments were confirmed by flow cytometry. All cells were cultured in either RPMI 1640 or Dulbecco's Modified Eagle Medium with 10% fetal bovine serum (FBS) (10270‐106; Gibco) and 1% penicillin/streptomycin at 37°C in an atmosphere of 5% CO_2_.

### 4.2 Generation and Isolation of RT‐EVs

Tumor cells (5 × 10^6^ cells/10 cm cell culture dish) were irradiated with a dose of 20 Gy by 6 MV X‐rays (600 MU/min; Trilogy System Linear Accelerator; Varian Medical Systems) and cultured with 20 mL complete medium. After 72 h, the medium was centrifuged at 1000 g for 10 min and then at 14 000 g for 2 min to remove cells and debris. The supernatant was centrifuged at 2000 g for 20 min to separate RT‐ABs, at 14 000 g for 60 min to isolate RT‐MPs, and at 200 000 g for 70 min to isolate RT‐EXOs. Following centrifugation, the RT‐EV precipitates were meticulously weighed.

To assess the role of apoptosis in RT‐MP generation, cells were pre‐treated with 40 µM Z‐VAD‐FMK (#HY‐16658B; MCE) 24 h prior to irradiation and the treatment was maintained for 72 h thereafter. EVs were then isolated following the standard extraction protocol described above.

### 4.3 Transmission Electron Microscopy

RT‐ABs, RT‐MPs, and RT‐EXOs were stained with 2% phosphotunstic acid solution for 5 min, then deposited on copper mesh. Transmission electron microscopy (HT7700‐SS/FEI Tecnai G20 TWIN) was used to observe the size and morphology.

### 4.4 Tumor Models and In Vivo Treatments

Male C57BL/6 mice (6–8 weeks) were purchased from Sikebas Laboratory (Henan, China). DTR/Zbtb46‐cre mice were obtained from Shanghai Model Organisms Center, Inc. (Shanghai, China). *Nlrp3^−/−^* mice and *Gsdmd^−/−^* mice were kindly gifted by professor Feng Shao (National Institute of Biological Sciences, Beijing). OT‐I mice were acquired from Shulaibao Biotechnology Co., Ltd (Wuhan, China). All animal experiments were performed according to protocols that were approved by the Hubei Provincial Animal Care and Use Committee (IACUC Number: 4333) and followed the experimental guidelines of the Animal Experimentation Ethics Committee of the Huazhong University of Science and Technology (HUST; Wuhan, China).

To establish the subcutaneous tumor implantation model, tumor cells (1 × 10^6^ cells/mouse) were subcutaneously injected into the right flank of each mouse. When the tumor volume reached about 30 mm^3^, mice were randomized to different groups and given treatment. For the control group, 200 µL 1 × PBS was intravenously injected, four times at 2 days intervals. For the RT‐EV groups, RT‐EVs (50 µg/mouse) were suspended in 200 µL 1 × PBS and intravenously injected four times at 2 days intervals. For the splenectomy group, mice were anesthetized by administration of 1% pentobarbital sodium before operation. The abdominal cavity was entered through a small incision under the left rib cage, splenic vessels were cauterized, and the spleen was removed two days before RT‐MP treatment. To evaluate the therapeutic efficacy of systemic IL‐1β administration, recombinant mouse IL‐1β (2 µg per mouse; #UA040072, UA·BIO) was administered via intraperitoneal injection every other day for a total of four doses. For the evaluation of intratumoral efficacy, RT‐MPs were administered via direct intratumoral injection. The particles (50 µg per mouse) were resuspended in 50 µL of 1× PBS and injected every other day, for a total of four doses. To evaluate the therapeutic efficacy of the treatments, subcutaneous tumor growth was recorded with the length (L) and width (W) of tumors by vernier calipers, and tumor size (V) was assessed using the formula V = (L × W^2^)/2. Mice were sacrificed when the tumor volume reached 1000 mm^3^.

To evaluate the role of endogenous RT‐MPs, tumor cells (2 × 10^6^ cells/mouse) were subcutaneously inoculated into the left flank of each mouse. When the tumor volume reached about 300 mm^3^, an additional inoculum of tumor cells (5 × 10⁵ cells/mouse) was administered subcutaneously into the right flank. Mice were then randomly assigned to the following treatment regimens: the control group received three intratumoral injections of 50 µL 1× PBS into the left tumor on alternate days; the Y27632 groups were given three intratumoral injections of Y27632 (HY‐10071; MCE, 10 mg/kg) into the left tumor on alternate days; the IR groups underwent a single 20 Gy irradiation of the left tumor using 6 MV X‐rays (600 MU/min; Trilogy System Linear Accelerator, Varian Medical Systems); and the combination (Y27632 + IR) groups received intratumoral Y27632 (10 mg/kg) one day before irradiation, followed by two additional injections on alternate days. Tumor growth on the right flank was monitored by measuring the length (L) and width (W) with vernier calipers, and tumor volume (V) was calculated as V = (L × W^2^)/2. Mice were euthanized when the volume of the left tumor reached 1000 mm^3^.

### 4.5 Near‐Infrared Fluorescence Imaging

To evaluate the influence of irradiation on EVs in vivo, Lewis cells were labeled with DIR and then subcutaneously injected (1 × 10^8^ cells/mouse) into the right flank of each mouse. After that, mice were separated into two groups at random. For the irradiation group, mice were irradiated with a dose of 20 Gy. After 24 h, spleens were collected and imaged by the Bruker In Vivo MS FX PRO Imager (ex: 740 nm; filter: 790 nm).

To detect the distribution of RT‐MPs in vivo, a subcutaneous xenograft tumor model of Lewis cells was established. When tumor volumes reached about 200 mm^3^, mice were separated into three groups at random. RT‐MPs were labeled with DIR and then intravenously injected into mice (50 µg/mouse). After 12 and 24 h, heart, liver, spleen, lung, kidney, tumor, right inguinal lymph nodes, and left inguinal lymph nodes were collected and imaged by the Bruker In Vivo MS FX PRO Imager.

### 4.6 Immune Cell Depletion

The depletion reagents were given starting on the day before RT‐MP treatment. The depletion efficacy was validated by flow cytometry.

In the DC depletion study, diphetheria toxin (List Biological Laboratories Inc.) was diluted in PBS and administered to DTR/Zbtb46‐cre mice via intraperitoneal injection (q3d ×3 doses, the first dose at 200 ng/mouse and the next two doses at 50 ng/mouse).

In the neutrophil depletion study, mouse anti‐Ly6G antibodies (#1A8; BioXCell) were diluted in PBS and administered via intraperitoneal injection (q3d ×3 doses, the first dose at 400 µg/mouse and the next two doses at 100 µg/mouse).

In the T cell depletion study, mouse anti‐CD8 antibodies (BE0061; BioXCell) or mouse anti‐CD4 antibodies (BE0003‐1; BioXCell) were diluted in PBS and administered via intraperitoneal injection (q3d ×3 doses, the first dose at 200 mg/mouse and the next two doses at 100 mg/mouse).

In the macrophage depletion study, clodronate liposomes (F70101CAC; FormuMax) were intraperitoneally injected (q3d ×3 doses, the first dose at 200 µL/mouse and the next two doses at 150 µL/mouse).

In the B cell depletion study, mouse anti‐CD19 antibodies (#C2849; Lenico) were intraperitoneally injected (q3d ×3 doses, the first dose at 75 µL/mouse and the next two doses at 50 µL/mouse).

In the NK cell depletion study, mouse anti‐NK1.1 antibodies (108701; BioLegend) were diluted in PBS and administered via intraperitoneal injection (q3d ×3 doses, 200 mg/mouse).

### 4.7 Multi‐Color Immunofluorescence Staining

For immunofluorescence labeling of CD8^+^ T cells, DCs, and neutrophils in mouse spleen, three doses of RT‐MPs were administered to C57BL/6 mice intravenously every other day. Spleens from control and RT‐MP‐treated mice were harvested 24 h after the last treatment. Samples were incubated with anti‐mouse CD8 (ab308264, ABcam), anti‐mouse CD11c (ab254183, ABcam), and anti‐mouse Ly6G (#87048, Cell Signaling Technology) antibodies. Tyramide signal amplification (TSA) Opal technology was used for multi‐immunofluorescence staining.

To visualize LC3 and N‐GSDMD localization, neutrophils were prepared as smears, fixed, and stained. The samples were probed with primary antibodies against LC3 (#GB11124‐50; Servicebio) and N‐GSDMD (#A24059; ABclonal).

### 4.8 Generation of BMDMs and BMDCs

Cells were collected from the bone marrow in femurs of C57BL/6 mice. The erythrocytes were removed by red blood cell lysis buffer. Subsequently, bone marrow single‐cell suspensions were cultured in RPMI 1640 medium supplemented with 10% FBS and 20 ng/mL GM‐CSF (315‐03; PeproTech) to generate BMDCs or 20 ng/mL M‐CSF (315‐02‐1MG; PeproTech) to generate BMDMs. The medium was replaced every 2 days. On the seventh day, BMDCs or BMDMs were harvested for assay.

### 4.9 Isolation of Neutrophils

To isolate neutrophils, peripheral blood and bone marrow in femurs of C57BL/6 mice was collected, followed by the lysis of erythrocytes. Neutrophils were isolated using MojoSort Mouse Neutrophil Isolation Kit according to the manufacturer's instructions.

### 4.10 Flow Cytometric Analysis

For assessing the influence of irradiation on EVs in vivo, Lewis cells were labeled with DIR and then subcutaneously injected (1 × 10^8^ cells/mouse) into the right flank of each mouse. After that, mice were separated into two groups at random. For the irradiation group, mice were irradiated with a dose of 20 Gy. After 24 h, spleens were collected, minced into pieces, gently ground, and filtered through a 40 mm strainer to obtain the single‐cell suspension. After the lysis of erythrocytes, the cell suspension was stained with Zombie Violet Fixable Viability Kit (#423113; BioLegend).

To detect the uptake of RT‐MPs in vivo, RT‐MPs were labeled with DIR and then intravenously injected into mice. After 4 and 24 h, peripheral blood and spleens were collected and prepared into single‐cell suspension. After the lysis of erythrocytes, the cell suspension was separated into two groups. The first group was stained with PerCP/Cy5.5‐anti‐CD45 (#157207; BioLegend), APC‐anti‐CD11c (#117309; BioLegend), PE/Cy7‐anti‐F4/80 (#123113; BioLegend), and BV421‐anti‐CD19 (#152421; BioLegend). The second group was stained with PerCP/Cy5.5‐anti‐CD45 (#157207; BioLegend), APC‐anti‐Ly6G (#127614; BioLegend), APC/Cy7‐anti‐Ly6C (#128026; BioLegend), and BV510‐anti‐CD3 (#100233; BioLegend).

To analyze changes in immune cells, the tumors were collected, minced into pieces, and digested with collagenase and hyaluronidase for 1 h at 37°C. Right inguinal lymph nodes and spleens were also collected and minced into pieces. Subsequently, the tissues were gently ground and filtered through a 40 mm strainer to obtain the single‐cell suspension followed by the lysis of erythrocytes.

To analyze DCs, cell suspensions were stained with Zombie NIR Fixable Viability Kit (#423106; BioLegend), FITC‐anti‐CD11c (#117305; BioLegend), BV421‐anti‐CD86 (#105031; BioLegend), APC‐anti‐CD80 (#104714; BioLegend), and PE/Cy7‐anti‐MHC‐II (#107629; BioLegend). To analyze T cells, a portion of cell suspensions was stimulated with Phorbol 12‐myristate 13‐acetate (100 ng/mL, ab120297, Abcam), Monensin sodium salt (1.5 mg/mL, ab120499, Abcam), and Ionomycin calcium salt (1 mg/mL, #5608212, PeproTech) for 6 h at 37°C with 5% CO_2_. Then, cell suspensions were stained with Zombie NIR Fixable Viability Kit (#423106; BioLegend), FITC‐anti‐CD3 (#100203; BioLegend), PE/Cy7‐anti‐CD4 (#100421; BioLegend), and BV510‐anti‐CD8a (#100751; BioLegend). Subsequently, cells were fixed and permeabilizated, then stained with BV421‐anti‐IFN‐γ (#505829; BioLegend), PE‐anti‐GzmB (#372207; BioLegend), and APC‐anti‐Foxp3 (#118903; BioLegend) in recommended antibody concentrations and incubated at 4°C for 30 min.

To assess the influence of RT‐MP‐treated neutrophils on DCs, the medium of RT‐MP‐treated neutrophils was collected and filtered with a 0.22 µm pore (MilliporeSigma), followed by the supplement with 10% FBS to prepare CM. Subsequently, DCs were treated with CM, with or without OVA peptide, RT‐MPs, and anti‐IL‐1β. Recombinant mouse IL‐1β (#UA040072, UA·BIO) was added to the DC cultures to a final concentration of 10 ng/mL. After that, DCs were stained with FITC‐anti‐CD11c (#117305; BioLegend), BV421‐anti‐CD86 (#105031; BioLegend), APC‐anti‐CD80 (#104714; BioLegend), PE/Cy7‐anti‐MHC‐II (#107629; BioLegend) and PE‐anti‐OVA‐SIINFEKL (#141611; BioLegend).

To determine whether RT‐MPs alter the expression of myeloid markers on Ly6G⁺ cells, cells treated with RT‐MPs were stained with anti‐CD115‐PE (#347303; BioLegend) and anti‐CD244‐FITC (#133504; BioLegend) and analyzed by flow cytometry.

### 4.11 Quantitative Inflammation Array

RT‐MPs were intravenously injected into C57BL/6. After 4 and 12 h, spleens were harvested for multiplex analysis of cytokines using Quantibody Mouse Inflammation Array 1 (QAM‐INF‐1; Raybiotech Inc.) according to the manufacturer's instructions.

### 4.12 Measurement of Lactate Dehydrogenase (LDH) Release

Neutrophils (5 × 10⁵ cells) were treated with RT‐MPs for 8 h, after which culture supernatants were collected. LDH activity in the supernatants was quantified using a commercial LDH assay kit (Beyotime) by measuring absorbance at 490 nm. The percentage of LDH release was calculated as follows: % LDH release = 100 × (experimental LDH − spontaneous LDH) / (maximum LDH release − spontaneous LDH).

### 4.13 ELISA

For spleen IL‐1β assays, RT‐MPs (50 µg/mouse) were suspended in 200 µL 1 × PBS and intravenously injected into C57BL/6. After 4 and 12 h, spleens were collected, lysed with RIPA buffer, and homogenized. The supernatant was collected and detected using Mouse IL‐1β Precoated ELISA Kit (1210122; DAKEWE) according to the manufacturer's instructions.

For neutrophil IL‐1β assays, neutrophils were treated with RT‐MPs for 6 h. The supernatant was collected for IL‐1β ELISA test. Neutrophils were treated with HSP90 inhibitor (Geldanamycin; MCE), STING inhibitor (C‐166; MCE), NF‐kB inhibitor (TPCA; MCE), the autophagy inhibitor 3‐MA (HY‐19312; MCE), or the pyroptosis inhibitor disulfiram (HY‐B0240; MCE) before RT‐MP treatments. RT‐MPs were incubated with DNase (Roche Diagnostics GmbH) or RNase (Beyotime Biotechnology) for DNA or RNA depletion.

### 4.14 Spleen Immune Cells Sorting and Single‐Cell mRNA‐seq Analysis

For the isolation of splenic neutrophils or Ly6G⁺ cells, spleens were harvested from tumor‐bearing mice and processed into single‐cell suspensions. Cells were stained with PerCP/Cy5.5‐conjugated anti‐CD45 (#157207, BioLegend) and APC‐conjugated anti‐Ly6G (#127614, BioLegend). CD45⁺Ly6G⁺ populations were sorted on a FACS sorter (MA900, SONY). The isolated cells were subsequently used for ELISA or flow cytometry analysis.

For scRNA‐seq analysis of immune cells in the spleen after RT‐MP treatments, three doses of RT‐MPs as mentioned above were administered to C57BL/6 mice. 24 h after the last administration, spleens were collected and ground into single‐cell suspension. Fresh samples were stained with Zombie NIR Fixable Viability Kit (#423106; BioLegend) and PerCP/Cy5.5‐anti‐CD45 (#157207; BioLegend) for subsequent FACS (MA900, SONY). Zombie^−^CD45^+^ cells were sorted as spleen immunocytes for immediate scRNA‐seq sample preparation.

Spleen immune cells were subjected to scRNA‐seq using MobiDrop (Zhejiang) Co., Ltd., China. Samples were loaded into the microfluidic chip of Chip a Single Cell Kit v2.1 (S050100301) to generate droplets with MobiNova‐100 (A1A40001). Each cell was encapsulated in a droplet containing a gel bead linked with millions of unique oligos (cell barcodes). Encapsulated droplets were cut with light by MobiNovaSP‐100 (A2A40001) while oligos diffused into the reaction mix. A unique cell barcode labeled mRNA with cDNA amplification in droplets. With a library built by the High Throughput Single‐Cell 3' Transcriptome Kit v2.1 (S050200301) and the 3' Dual Index Kit (S050300301), sequencing was conducted on an Illumina NovaSeq 6000 System.

Mus musculus reference GRCm39 was used for FASTQ files initial processing by MobiVision software (version 2.1). Unique molecular identifier (UMI) counts were then aggregated for each barcode, and the count matrix was analyzed using the Seurat R package (version 4.0.0). Poor‐quality cells and potential multiple captures were discarded by the following criteria: gene numbers < 200, UMI < 1000, log10GenesPerUMI < 0.7, proportion of UMIs mapped to mitochondrial genes > 10%, and proportion of UMIs mapped to hemoglobin genes >5%. Subsequently, we used the DoubletFinder package (version 2.0.3) to identify potential doublets and multiplets and the NormalizeData function to normalize library size. Normalization of gene expression for each cell was conducted under “LogNormalize,” which involved multiplying the total expression by a scaling factor (default is 10 000) and then log‐transforming the results.

Cells were clustered by gene expression using the FindClusters function. A 2‐dimensional Uniform Manifold Approximation and Projection (UMAP) algorithm was used for visualization with the RunUMAP function. Marker genes for each cluster were identified using the FindAllMarkers function (test. use = presto). Differentially expressed genes (DEGs) were selected using the FindMarkers function (test. use = presto), with a significance threshold of *p* value < 0.05 and |log2foldchange| > 0.58. GO enrichment and KEGG pathway enrichment analyses of DEGs were conducted using R (version 4.0.3) based on the hypergeometric distribution.

### 4.15 RNA‐Seq

Neutrophils were treated with or without RT‐MPs for 6 h, after which they were washed twice with PBS and centrifuged at 1000 g for 10 min. The cells were rapidly frozen in Trizol reagent at ‐80°C. Then, samples were sent to Biomarker Technologies Co., Ltd. (Beijing, China).

### 4.16 Quantitative Real‐Time PCR

Neutrophils were treated with or without RT‐MPs for 6 h, after which they were washed twice with PBS, and centrifuged at 1000 g for 10 min. RNA was extracted using the Total RNA Kit I R6834 (Omega), and was measured using the NanoDrop ND‐1000 (Thermo Fisher Scientific). Purified RNA was reverse‐transcribed into complementary DNA with HiScript III RT SuperMix (+gDNA wiper) (Vazyme), according to the manufacturer's protocol. qRT‐PCR reactions were performed in the StepOnePlus Real‐Time PCR system using ACEQ Universal SYBR qPCR Master Mix (Vazyme). GAPDH expression was used for normalization. The sequences of the primers used are listed in Table S1. All primers in the study were synthesized by GENE CREATE.

### 4.17 Amplification and Visualization of mtDNA in RT‐MPs

DNA in RT‐MPs was obtained under the instructions of DNA extraction kit (Beyotime Biotechnology). Murine mtDNA was amplified by 14 pairs of primers (1 ng DNA per pair of primers) designed by mtDNA‐specifically encoded genes (Table S2). Amplicons were visualized on a 1% agarose gel under UV exposure.

### 4.18 Western Blot

Protein samples from cells were prepared with RIPA lysis buffer and separated by SDS‐PAGE, followed by transferring to polyvinylidene fluoride membranes. After blocked in 5% skim milk powder dissolved in Tris‐buffered saline containing 0.1% Tween 20 at room temperature for 1 h, the membranes were incubated with corresponding primary antibodies overnight at 4°C. After washing and incubation with diluted secondary antibody conjugated to horseradish peroxidase, the membranes were soaked with NcmECL Ultra (P10100; NCM Biotech) and imaged with an infrared imaging system (UPV ChemiDoc‐It 510, Thermo Fisher Scientific). The primary antibodies were listed as follows: anti‐NLRP3 (#30109‐1‐AP; Proteintech), anti‐Caspase11 (#67398‐1‐Ig; Proteintech), anti‐GSDMD (#39754; Cell Signaling Technology), anti‐STING (#13647; Cell Signaling Technology), anti‐phospho STING S366 (#AP1369; ABclonal) and GAPDH (#60004‐1‐Ig; Proteintech), anti‐CD9 (#20597‐1‐AP; Proteintech), anti‐CD63 (#25682‐1‐AP; Proteintech), anti‐TSG101 (#28283‐1‐AP; Proteintech), anti‐ARF6 (#A11485; ABclonal), and anti‐KIF23 (#28587‐1‐AP; Proteintech).

### 4.19 T Cell Cytotoxicity Assay

DCs were treated with CM, with or without RT‐MPs and anti‐IL‐1β. CD8^+^ T cells were isolated from the spleens of OT‐I mice, and following isolation, these cells were incubated with DCs in different groups for 24 h. The assessment of cancer cell sensitivity to T cell‐mediated cytotoxicity involved staining Lewis‐OVA with Calcein‐AM at a final concentration of 50 µm. The staining was ended with wash by cell culture containing 10% FBS, twice. Then, T cells were added at E: T ratios of 5:1. After an 8‐h incubation, the co‐culture in 96‐well plates was centrifuged, and the Calcein‐AM fluorescence intensity of supernatant was measured using fluorescence microplate reader at an excitation wavelength of 490 nm, determining the cytotoxic efficiency of the CD8^+^ T cells. The percentage of specific lysis was calculated as follows: % specific lysis = [(experimental sample release—effector cells spontaneous control—target cells spontaneous control)/(maximum release—target cells spontaneous control)] × 100%.

### 4.20 Statistical Analysis

Statistical analysis was performed using Prism 9.0 software (GraphPad Software). For comparisons of two groups, an unpaired two‐tailed Student's *t*‐test was performed. One‐way ANOVA was performed on multiple groups. Tumor growth was assessed using Two‐way ANOVA. *p*‐values of <0.05 were considered to indicate statistical significance (*p < 0.05, **p < 0.01, and ***p < 0.001). Data are presented as mean ± standard error of the mean.

## Author Contributions

Y.H., J.W., C.W., and Y.S. designed the study and wrote the manuscript. Y.H., J.W., M.C., and Y.S. performed experiments. Y.H., J.W., C.W., Y.S., M.C., Z.Y., J.M., X.Y., Y.D., Z.Z., W.W., and Y.W. analyzed and discussed the data. J.Z., B.W., Y.Q., H.J., and F.H. provided the advisement. C.W., L.W., Y.S., and K.Y. supervised the research and revised the manuscript.

## Conflicts of Interest

K.Y., H.J., Y.S., and C.W. are inventors on the patent related to this work filed by Union Hospital, Tongji Medical College, Huazhong University of Science and Technology (no. 2019100311342, 14 January 2019). The other authors declare that they have no competing interests.

## Supporting information




**Supporting File**: advs73689‐sup‐0001‐SuppMat.docx.

## Data Availability

The data that support the findings of this study are available in the supplementary material of this article.
