# Supplementary material for: Irradiated Tumor Cell‐Derived Microparticles Activate Systemic Anti‐Tumor Immunity via the STING/NLRP3/GSDMD Axis in Neutrophils
Source: Adv Sci (Weinh). 2026 Jan 4;13(16):e14390. doi: 10.1002/advs.202514390 (PMC13042607; doi:10.1002/advs.202514390)
Supplement: Supplementary file 1 — Supporting File: advs73689‐sup‐0001‐SuppMat.docx. [file ADVS-13-e14390-s001.docx]

**Supplemental Material**

**Irradiated Tumor Cell-Derived Microparticles Activate Systemic Anti-Tumor Immunity via the STING/NLRP3/GSDMD Axis in Neutrophils**

Yan Hu^1,2,3,4#^; Jiacheng Wang^1,2,3,4#^; Mengjie Che^1,2,3,4#^; Zheng Yang^1,2,3,4^; Jingshu Meng^1,2,3,4^; Xiao Yang^1,2,3,4^; Yue Deng^1,2,3,4^; Zhiyuan Zhou^1,2,3,4^; Yijun Wang^1,2,3,4^; Wenwen Wei^1,2,3,4^; Zhanjie Zhang^1,2,3,4^; Bian Wu^1,2,3,4^; You Qin^1,2,3,4^; Kunyu Yang^1,2,3,4^; Honglin Jin^5^; Fang Huang^1,2,3,4^; Yajie Sun^1,2,3,4^*; Lu Wen^1,2,3,4^*; Chao Wan^1,2,3,4^*

1. Cancer Center, Union Hospital, Tongji Medical College, Huazhong University of Science and Technology, Wuhan 430022, China

2. Institute of Radiation Oncology, Union Hospital, Tongji Medical College, Huazhong University of Science and Technology, Wuhan 430022, China

3. Hubei Key Laboratory of Precision Radiation Oncology, Wuhan 430022, China

4. Key Laboratory of Biological Targeted Therapy (Huazhong University of Science and Technology), Ministry of Education, Wuhan, Hubei 430022, China

5. College of Biomedicine and Health and College of Life Science and Technology, Huazhong Agricultural University, Wuhan 430070, China

^#^These authors have contributed equally to this article.

*Correspondence: wanc@hust.edu.cn; wenlu2808@126.com; sunyajie@hust.edu.cn


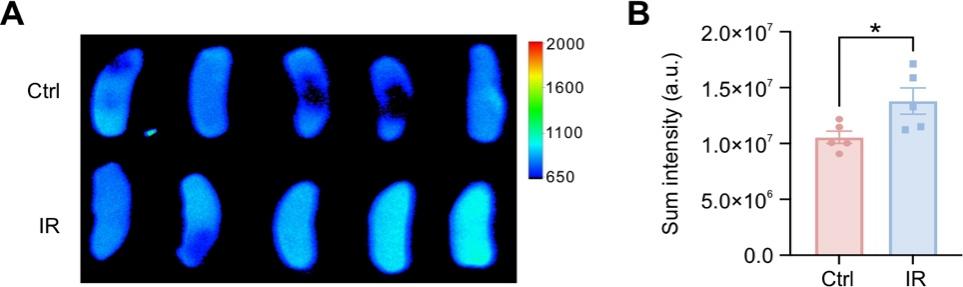


**Figure S1. Irradiation enhances the accumulation of RT-EVs in the spleen.**

(A) Near-infrared fluorescence imaging of DIR signals in spleens. (B) Fluorescence intensities of the spleen as evaluated by quantitative analysis (n = 5). *p < 0.05, **p < 0.01, and ***p < 0.001. Data are presented as mean ± SEM; two-tailed unpaired t-test for (B).


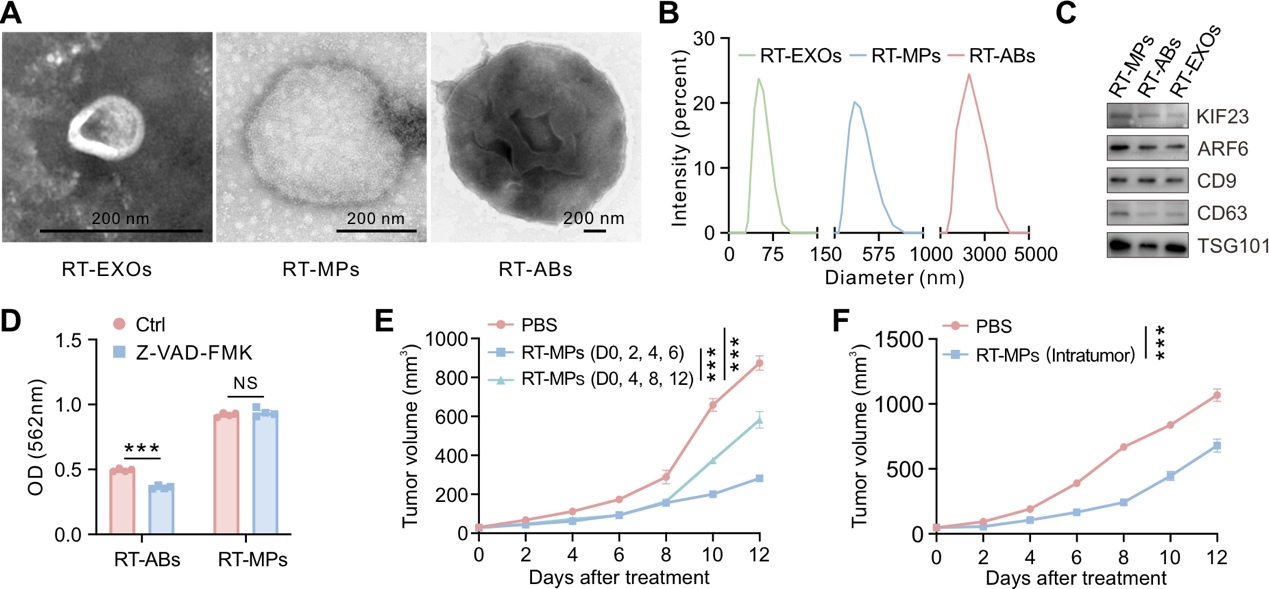


**Figure S2. Characterization of RT-EVs and the antitumor effect of RT-MPs under different administration regimens.**

(A) TEM images of RT-EXOs, RT-MPs, and RT-ABs (scale bar, 200 nm). (B) Representative NTA size distributions of RT-EXOs, RT-MPs, and RT-ABs. (C) Western blot analysis of canonical EV markers (KIF23, ARF6, CD9, CD63, and TSG101) in RT-EV (n = 3). (D) Yields of RT-MPs and RT-ABs following treatment with the pan-caspase inhibitor Z-VAD-FMK, quantified by BCA protein assay (n = 4). (E) Tumor growth kinetics following RT-MPs treatment administered at different time intervals (n = 6-7). (F) Tumor growth curves after intratumoral injection of RT-MPs in the Lewis lung carcinoma subcutaneous model (n = 8). *p < 0.05, **p < 0.01, and ***p < 0.001. Data are presented as mean ± SEM; two-tailed unpaired t-test for (D); two-way ANOVA for (E, F).


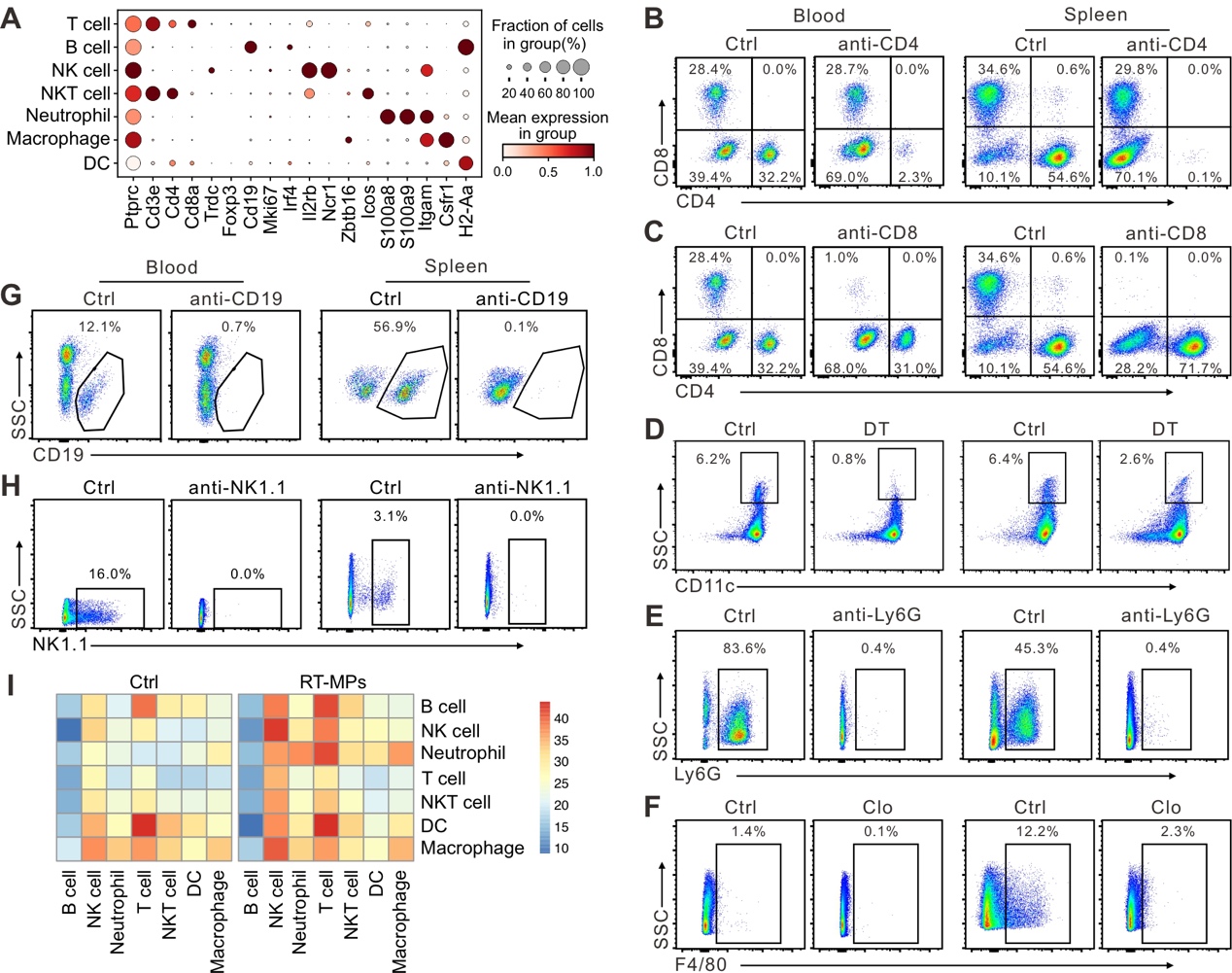


**Figure S3. Characterization of CD45^+^ immune cell clusters in the spleen and validation of immune cell depletion efficiency, related to Figure 3.**

(A) Bubble heatmap showing selected genes in CD45^+^ immune cell clusters. (B-H) The depletion of immune cells in the blood and spleen. Flow cytometry analysis of anti-CD4-mediated depletion efficiency in CD4^+^ T cells (B), anti-CD8-mediated depletion efficiency in CD8^+^ T cells (C), DT-mediated depletion efficiency in DCs (D), anti-Ly6G-mediated depletion efficiency in neutrophils (E), Clo-mediated depletion efficiency in macrophages (F), anti-CD19-mediated depletion efficiency in B cells (G), and anti-NK1.1-mediated depletion efficiency in NK cells (H). (I) Heatmap showing the crosstalk between immune cell types. The color bar represents the number of ligand-receptor pairs.


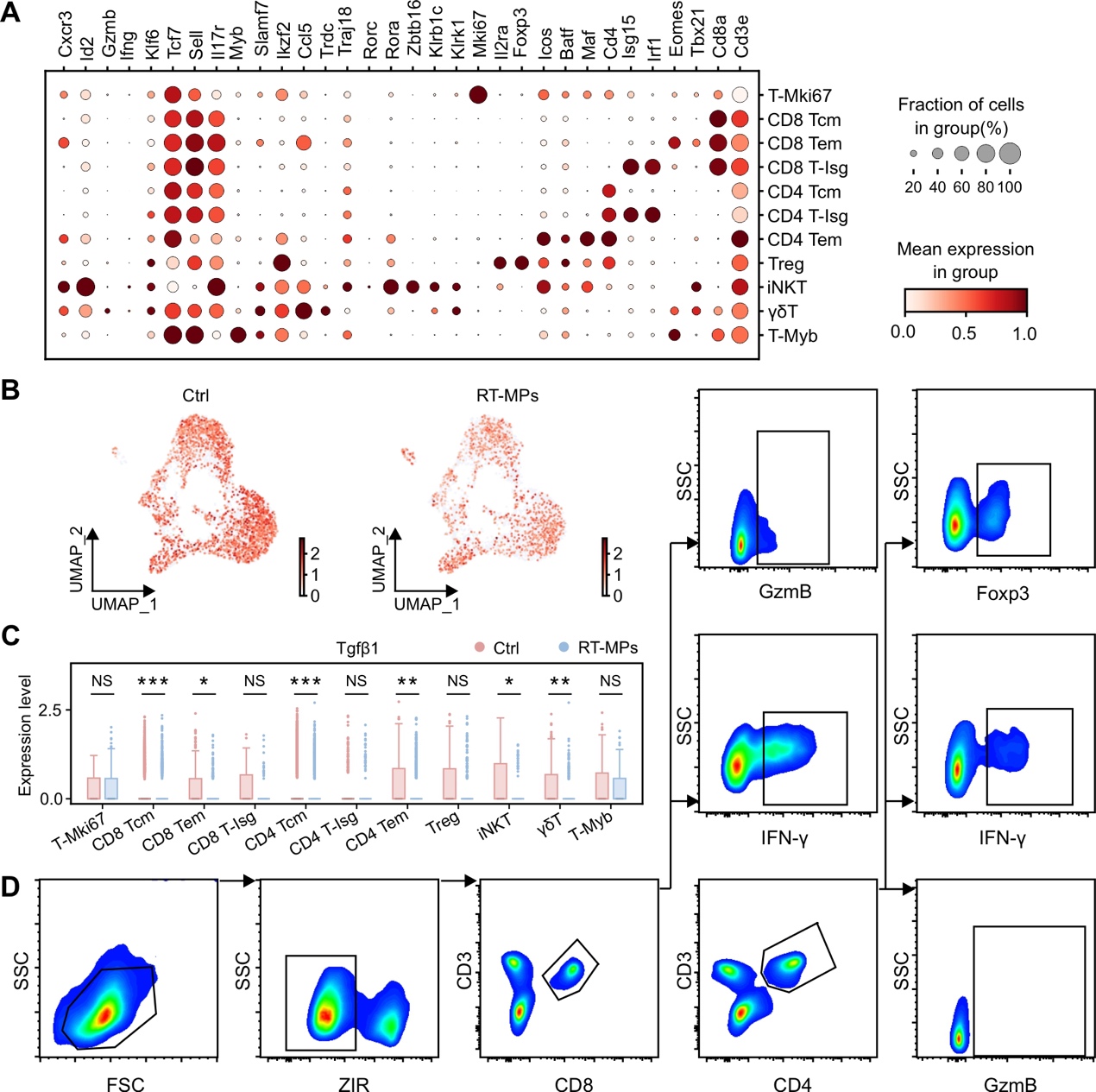


**Figure S4. Characterization of T cell clusters in the spleen, related to Figure 4.**

(A) Bubble heatmap showing selected genes in T cell clusters. (B) UMAP plot of *Tgfβ1* expression in T cells as described in (A). (C) Comparison of *Tgfβ1* expression levels in each T cell cluster. (D) Flow cytometry gating strategy for the T cell detection. *p < 0.05, **p < 0.01, and ***p < 0.001. Data are presented as mean ± SEM; two-tailed unpaired t-test for (C).

**
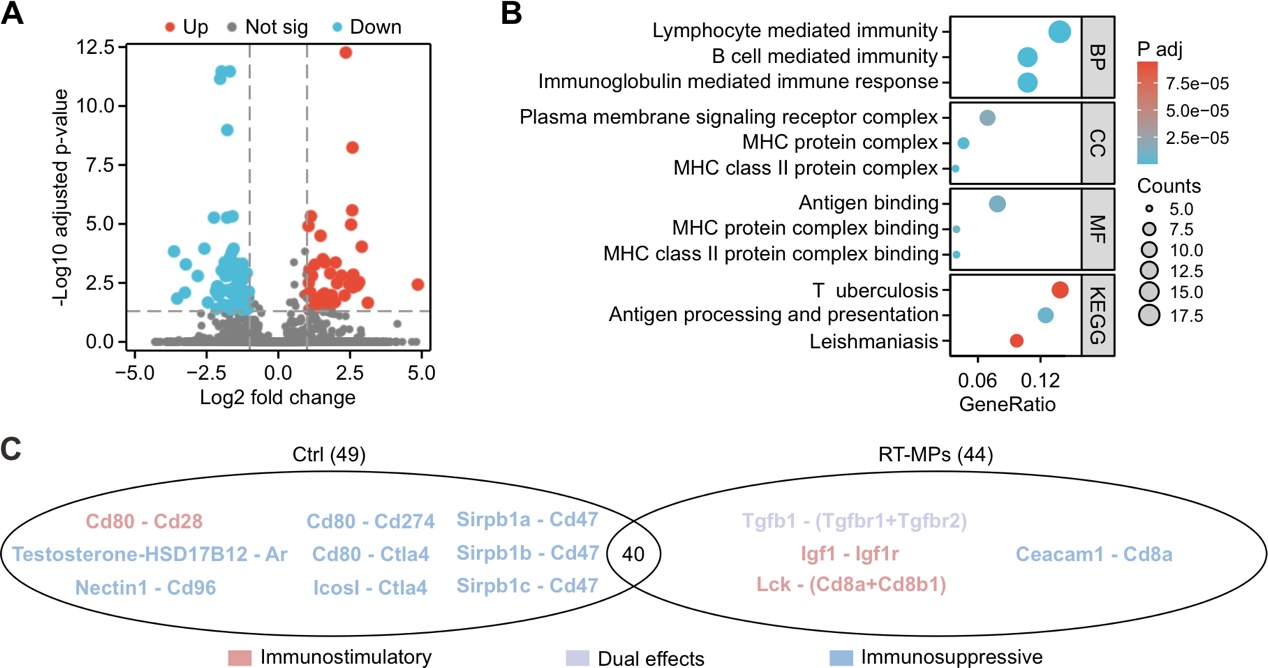
**

**Figure S5. Differentially expressed genes of DCs in the spleen, related to Figure 5.** (A) Volcano plot showing differentially expressed genes of DCs in the spleen from RT-MP-treated mice. (B) GO enrichment analysis and KEGG enrichment analysis of differentially expressed genes for DCs in the spleen from RT-MP-treated mice. (C) The significantly enriched ligand-receptor pairs between DCs and T cells.

**
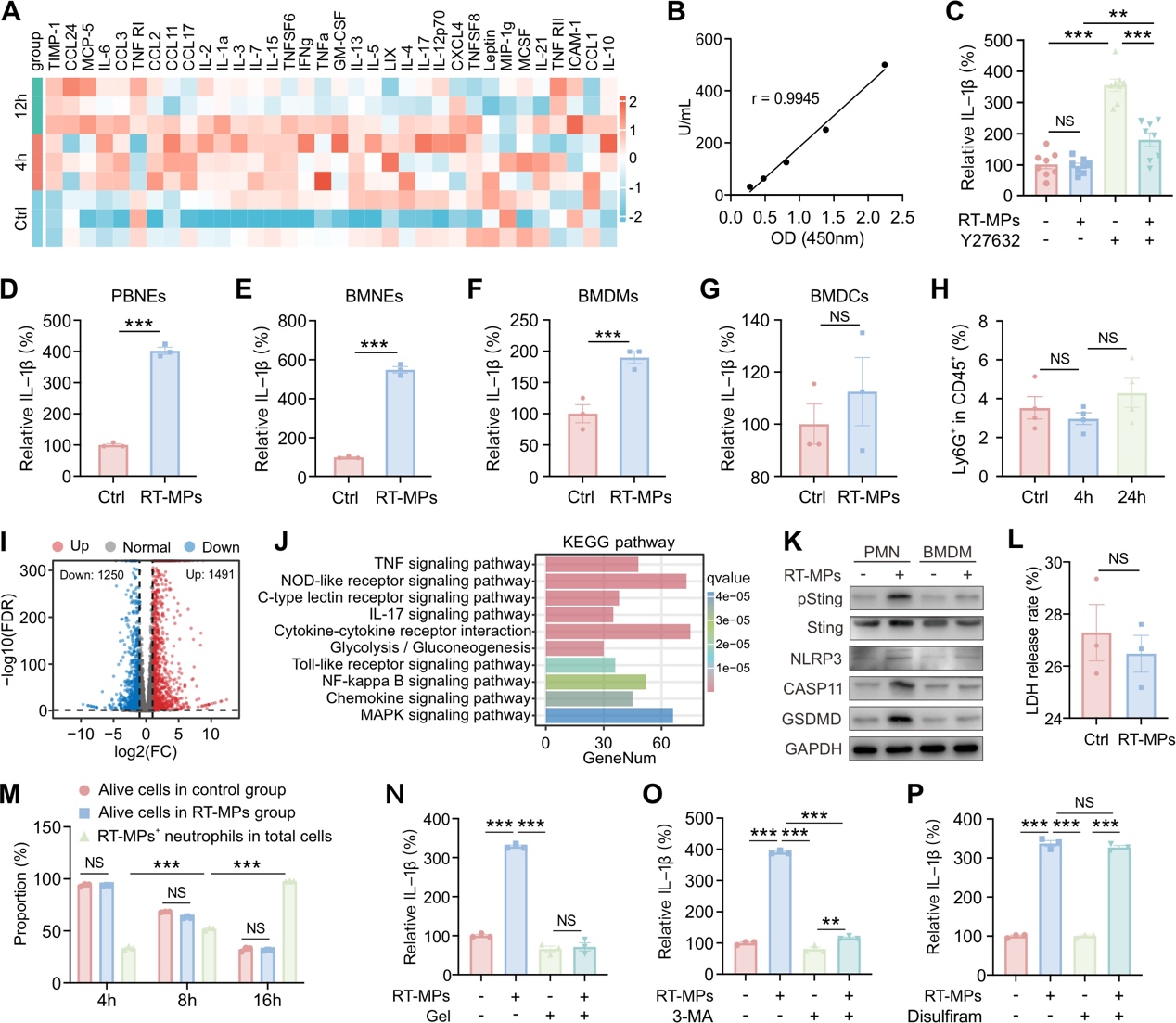
**

**Figure S6. RT-MPs promote** **IL-1β release from neutrophils, related to Figure 6.**

(A) Heatmap of cytokine array results in the spleen from the corresponding treatment groups (n = 3). (B) Standard curve for detecting IL-1β. (C) Splenic IL-1β levels following radiotherapy and *in vivo* inhibition of vesicle release by Y27632, measured by ELISA (n = 8). (D-E) IL-1β levels in culture supernatants of peripheral blood neutrophils (PBNEs; D) and bone marrow neutrophils (BMNEs; E) treated with RT-MPs, measured by ELISA (n = 3). (F-G) IL-1β levels in culture supernatants of BMDMs (F) and BMDCs (G) treated with RT-MPs, measured by ELISA (n = 3). (H) Proportion of neutrophils in the spleen after RT-MPs treatment, determined by flow cytometry (n = 4). (I) Volcano plot showing differentially expressed genes between PBS-treated and RT-MP-treated neutrophils. (J) The top 10 upregulated functional pathways in PBS-treated and RT-MP-treated neutrophils, as determined by KEGG analysis. (K) Western blots of pSTING, STING, NLRP3, CASP11, GSDMD, and GAPDH expression in both neutrophils (PMNs) and BMDMs treated with RT-MPs (n = 3). (L) LDH release in the culture media of neutrophils following RT-MPs treatment (n = 3). (M) Quantification by flow cytometry of viable neutrophils and PKH26⁺ neutrophils (internalized RT‑MPs) over time (n = 3). (N) IL-1β levels in the culture media of RT-MP-treated neutrophils upon co-treatment with the Hsp90 inhibitor Geldanamycin (Gel), measured by ELISA (n = 3). (O, P) Effect of the autophagy inhibitor 3‑methyladenine (3‑MA) (O) or the pyroptosis inhibitor disulfiram (P) on IL‑1β secretion from RT‑MP‑treated neutrophils, as quantified by ELISA (n = 3). *p < 0.05, **p < 0.01, and ***p < 0.001. Data are presented as mean ± SEM; two-tailed unpaired t-test for (D-G, L, M); one-way ANOVA for (C, H, O, P).

**
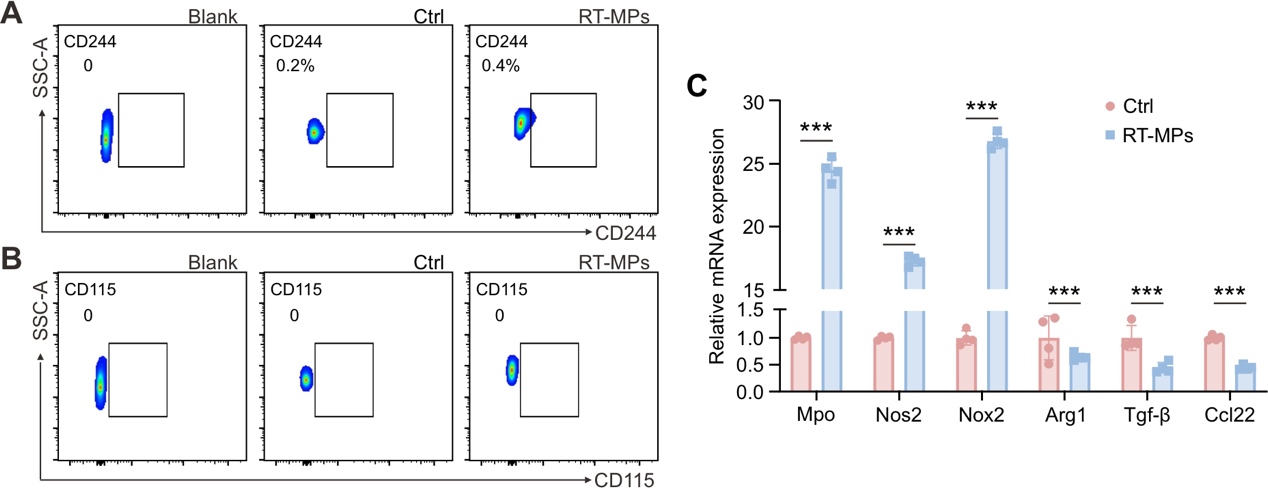
**

**Figure S7. RT-MPs promote a pro-inflammatory phenotype in Ly6G^+^ cells without upregulating canonical myeloid-derived suppressor cells (MDSCs) markers.**

(A-B) Representative images of CD244 (A) and CD115 (B) expression in splenic Ly6G^+^ cells sorted from Lewis-tumor bearing mouse. (C) Transcriptional levels of *Mpo*, *Nos2*, *Nox2*, *Arg1*, *Tgf-β*, and *Ccl22* in splenic neutrophils after RT-MPs stimulation (n = 4). *p < 0.05, **p < 0.01, and ***p < 0.001. Data are presented as mean ± SEM; two-tailed unpaired t-test for (C).


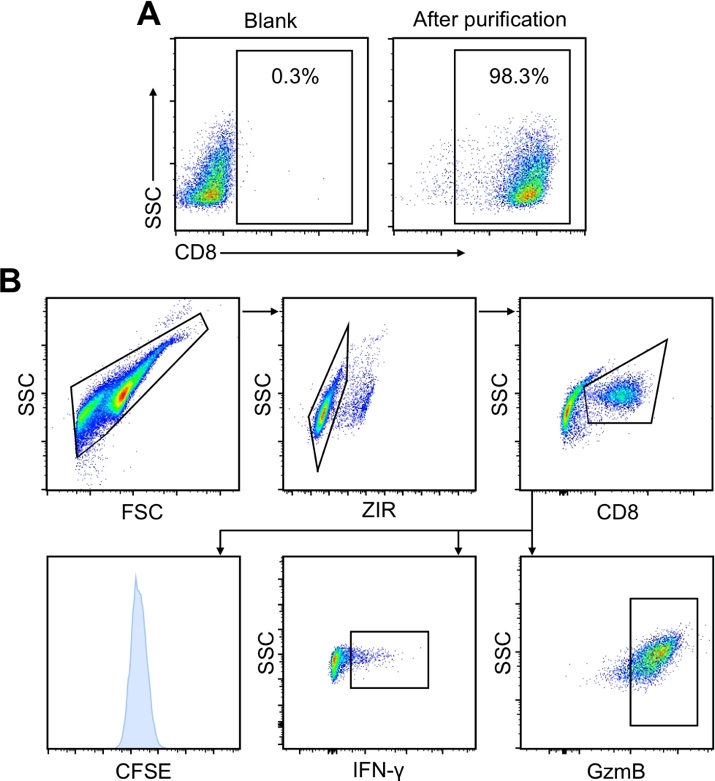


**Figure S8. Cell sorting efficiency of CD8^+^ T cells and flow cytometry gating strategy for the detection of T cells, related to Figure 7.**

(A) Flow cytometry was used to assess the cell sorting efficiency of CD8^+^ T cells from OT-I mice. (B) Flow cytometry gating strategy for T cell detection.

**
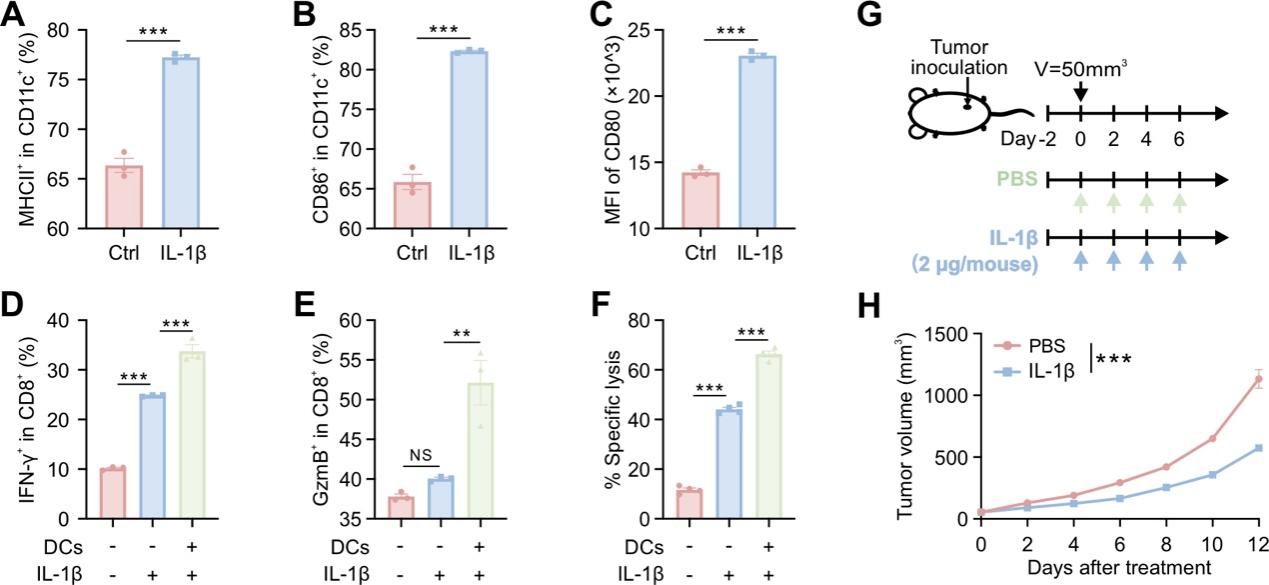
**

**Figure S9. IL-1β inhibits tumor growth through enhancing DC-mediated T cell activation.**

(A-C) Flow cytometric analysis of MHC-II (A), CD86 (B), and CD80 (C) expression in DCs following IL-1β treatment (n = 3). (D-E) Expression of IFN-γ (D) and GzmB (E) in CD8⁺ T cells after IL-1β treatment, as determined by flow cytometry (n = 3). (F) Specific lysis of Lewis-OVA tumor cells by OT-I T cells that were pretreated with the indicated DCs under IL-1β conditioning (n = 4). (G) Scheme of the IL-1β treatment regimen. (H) Tumor growth kinetics in the Lewis lung carcinoma subcutaneous model following IL-1β treatment (n = 7). *p < 0.05, **p < 0.01, and ***p < 0.001. Data are presented as mean ± SEM; two-tailed unpaired t-test for (A-C); one-way ANOVA for (D-F); two-way ANOVA for (H).

**Table S1. Sequences of primers used for qRT-PCR.**

| Genes | Sequences (5’--3’) |
| --- | --- |
| Nlrp3 | F: ATCAACAGGCGAGACCTCTG |
|  | R: GTCCTCCTGGCATACCATAGA |
| Casp11 | F: TGCCTTCTACTCTACAACC |
|  | R: CTGGGAATGAATACTTGC |
| Gsdmd | F: TTCCAGTGCCTCCATGAATGT |
|  | R: GCTGTGGACCTCAGTGATCT |
| Pro Il-1b | F: TTCAGGCAGGCAGTATCACTC |
|  | R: GAAGGTCCACGGGAAAGACAC |
| Nos2 | F:GTTCTCAGCCCAACAATACAAGA |
|  | R:GTGGACGGGTCGATGTCAC |
| Mpo | F:GAGTCCCACTCAGCAAGGTC |
|  | R:TCTGGCGATTCAGTTTGGCT |
| Nox2 | F:CCAGTGAAGATGTGTTCAGCT |
|  | R:GCACAGCCAGTAGAAGTAGAT |
| Arg1 | F:GGTTCTGGGAGGCCTATCTT |
|  | R:CACCTCCTCTGCTGTCTTCC |
| Tgf-β | F:AACAATTCCTGGCGTTACCT |
|  | R:GGCTGATCCCGTTGATTTCC |
| Ccl22 | F: CAGGACTACATCCGTCACCC |
|  | R: TGAGTAAAGGTGGCGTCGTT |
| Gapdh | F: AGGTCGGTGTGAACGGATTTG |
|  | R: TGTAGACCATGTAGTTGAGGTCA |

F, forward primer; R, reverse primer.

**Table S2. Sequences of primers used for murine mtDNA amplification.**

| Murine mtDNA Genes | Sequences (5’--3’) |
| --- | --- |
| DLoop | F: AGGTTTGGTCCTGGCCTTAT |
|  | R: GTGGCTAGGCAAGGTGTCTT |
| ND1(1) | F: CTAGAAACCCCGAACCAAA |
|  | R: CCAGCTATCACCAAGCTCGT |
| ND1(2) | F: CAGCCGGCCCATTCGCGTTA |
|  | R: AGCGGAAGCGTGGATAGGATGC |
| ND2 | F: TCCTCCTGGCCATCGTACTCAACT |
|  | R: AGAAGTGGAATGGGGCGAGGC |
| ND4L | F: TCGCTCCCACCTAATATCCACATTGC |
|  | R: GCAGGCTGCGAAAACCAAGATGG |
| ND4 | F: TCGCCTACTCCTCAGTTAGCCACA |
|  | R: TGATGATGTGAGGCCATGTGCGA |
| ND5 | F: TCGGAAGCCTCGCCCTCACA |
|  | R: AGTAGGGCTCAGGCGTTGGTGT |
| ND6 | F: AATACCCGCAAACAAAGATCACCCAG |
|  | R: TGTTGGGGTTATGTTAGAGGGAGGGA |
| COX1 | F: CCAGTGCTAGCCGCAGGCAT |
|  | R: TCTGGGTGCCCAAAGAATCAGAACA |
| COX2 | F: AGTTGATAACCGAGTCGTTCTGCCA |
|  | R: TCGGCCTGGGATGGCATCAGT |
| COX3 | F: ACCTACCAAGGCCACCACACTCC |
|  | R: GCAGCCTCCTAGATCATGTGTTGGT |
| ATP8 | F: ATGCCACAACTAGATACATCAACA |
|  | R: GGGGTAATGAATGAGGCAAA |
| ATP6 | F: GCTCTCACTCGCCCACTTCCTTCC |
|  | R: GCCGGACTGCTAATGCCATTGGTT |
| Cytb | F: ACAGCAAACGGAGCCTCAA |
|  | R: TGCTGTGGCTATGACTGCGAACA |

F, forward primer; R, reverse primer.
